# Molecular evolution and diversification of phytoene synthase (PSY) gene family

**DOI:** 10.1590/1678-4685-GMB-2021-0411

**Published:** 2022-12-19

**Authors:** Marcia Pagno Lisboa, Drielli Canal, João Pedro Carmo Filgueiras, Andreia Carina Turchetto-Zolet

**Affiliations:** 1 Universidade Federal do Rio Grande do Sul (UFRGS), Departamento de Genética, Instituto de Biociências, Programa de Pós-Graduação em Genética e Biologia Molecular, Porto Alegre, RS, Brazil.

**Keywords:** biosynthesis, evolutionary history, expression profile, paralogs, phylogeny

## Abstract

Phytoene synthase (PSY) is a crucial enzyme required for carotenoid biosynthesis, encoded by a gene family conserved in carotenoid-producing organisms. This gene family is diversified in angiosperms through distinct duplication events. Understanding diversification patterns and the evolutionary history of the PSY gene family is important for explaining carotenogenesis in different plant tissues. This study identified 351 PSY genes in 166 species, including Viridiplantae, brown and red algae, cyanobacteria, fungi, arthropods, and bacteria. All PSY genes displayed conserved intron/exon organization. Fungi and arthropod PSY sequences were grouped with prokaryote PSY, suggesting the occurrence of horizontal gene transfer. Angiosperm PSY is split into five subgroups. One includes the putative ortholog of PSY3 (Subgroup E3) from eudicots, and the other four subgroups include PSY from both monocots and eudicots (subgroups E1, E2, M1, and M2). Expression profile analysis revealed that PSY genes are constitutively expressed across developmental stages and anatomical parts, except for the eudicot PSY3, with root-specific expression. This study elucidates the molecular evolution and diversification of the PSY gene family, furthering our understanding of variations in carotenogenesis.

## Introduction

Carotenoids are a complex class of C40 isoprenoid pigments synthesized by photosynthetic organisms, non-photosynthetic bacteria, and fungi (Cuttriss et al., 2011). In the chloroplast, carotenoids participate in photosynthesis, contribute to photoprotection, and act as precursors for strigolactone and abscisic acid (ABA) biosynthesis. They thus play a major role in mediating developmental signaling and stress responses. As secondary metabolites, carotenoids accumulate in chromoplasts, providing attractive colors and aroma precursors in fruits and flowers essential for pollination and seed dispersal (Ahrazem et al., 2019). 

Except for some arthropods (Moran and Jarvik, 2010; Grbić et al., 2011; Cobbs et al., 2013), animals are unable to synthesize carotenoids *de novo*. Instead, the compounds are obtained directly from food consumed or partially modified through metabolic reactions (Maoka, 2019). Carotenoids are essential dietary nutrients metabolized to retinol (vitamin A) and its derivatives. Retinol oxidation provides retinal, necessary for vision, and retinoic acid, a transcription factor ligand essential to regulating genes involved in cell morphogenesis, differentiation, and proliferation (Dawson, 2000). 

Carotenoid biosynthesis in plants is well described. Phytoene synthase (PSY) converts two molecules of geranylgeranyl pyrophosphate (GGPP) (C20) into phytoene (C40). Phytoene desaturase (PDS) then catalyzes colorless phytoene into lycopene, forming 9, 15, 9-tri-cis-ζ-carotene. The product is then desaturated via ζ-carotene desaturase (ZDS) to generate 7, 9, 9, 7-tetra-cis-lycopene (prolycopene), and from here, subsequent reactions are responsible for producing the different carotenoids found in nature (Nakkanong et al., 2012). Carotenoid color varies according to the number of double bonds, with greater unsaturation corresponding to shorter absorbed wavelengths. Phytoene and phytofluene carotenoids are colorless, zeta-carotene is yellow, neurosporene is yellowish-orange, and lycopene is red. During desaturation, various intermediate reactions with the *cis* configuration are produced (Tanaka et al., 2008). Phytoene synthase is pivotal to the carotenoid pathway as the first committed biosynthetic step, controlling metabolic flux through the pathway (Welsch et al., 2000). 

Previous analysis of carotenoid pathway genes indicated early evolutionary roots in prokaryotes, with more than 700 different natural carotenoid structures identified, many presents in bacteria. Genes encoding C40 phytoene are well conserved in Archaea and bacteria, indicating a common carotenogenic progenitor (Sandmann, 2021). In plants, two *PSY* genes (*PSY1* and *PSY2*) are present inthe angiosperm ancestor and a specific duplication event in both the monocot (Dibari et al., 2012) and eudicot (Han et al., 2015; Stauder et al., 2018) groups resulted in a third *PSY* (*PSY3*) gene. *PSY3* seems to have evolved independently in the monocot and eudicot species. Most plant species have a *PSY* gene family comprising two or three homologous genes. *Arabidopsis* has a single *PSY* gene, whereas carrots have two *PSY* genes (Clotault et al., 2008; Rodriguez-Villalon et al., 2009). Tomato, cassava, and grasses such as maize, rice, and sorghum have three *PSY* paralogs in their genomes (Chaudhary et al., 2010; Dibari *et al*., 2012). *PSY* gene duplication has led to subfunctionalization, with each paralog exhibiting differential gene expression. This functional diversification of *PSY* homologs allows carotenoids to accumulate in non-photosynthetic tissues (e.g., fruits, seeds, and flowers) and respond to environmental stress (Cárdenas et al., 2012).

Thus, in this work, we inferred a phylogenetic tree including *PSY* genes found in complete genomes of a range of taxa, including bacteria, algae, plants, arthropods, and fungi. We then mapped the possible *PSY* duplication and loss events on the tree’s internal nodes. Finally, we investigated the functional divergence of the *PSY* paralogs. This analysis allowed us to explore the protein motif and domain organization, gene structure, and expression patterns of *PSY* genes in different tissues. This study contributes to understanding *PSY* gene family evolution and functional divergence.

## Material and Methods

### Database search and sequence retrieval

*PSY* homologs were identified via BLAST searches in public databases (NCBI, Phytozome 12.1, Ensembl Plants, Congenie, and *Klebsormidium* Genome Project). Query sequences were selected from organisms in which *PSY* was previously identified and characterized. A preliminary BLASTp search was performed with three *PSY* sequences identified in *Solanum lycopersicum* (Solyc03g031860.2.1, Solyc02g081330.2.1, and Solyc01g005940.2.1) (Giorio et al., 2008; Stauder et al., 2018), and the results indicated that each recovered practically the same sequence. Thus, only Solyc03g031860.2.1 was used for a BLASTp search (with default parameters) against 63 fully sequenced genomes of Viridiplantae species from  Phytozome; 28 Viridiplantae species from Ensembl Plants; 13 Rhodophyta species, 12 Cyanobacteria species, 11 Ochorophyta species, 10 Fungi species, and six Arthropoda species from NCBI; nine Prokaryote species from Ensembl Bacteria; two Gymnosperm species from Congenie; and one representative Charophyta species from Plant morphogenesis. Additionally, the *Pantoea ananatis* (D90087.2) sequence was used for a BLASTp search with default parameters against 10 Prokaryotespeciesfrom NCBI. The *Tetranychus urticae* (tetur01g11260) sequence was used for a BLASTp search with default parameters against one Arthropoda species from Ensembl Metazoa. These searches yielded166 species for analysis, and 351 sequences were retrieved (Table S1).

### Sequence alignment and phylogenetic analysis

Protein sequences were aligned using MUSCLE (Edgar, 2004) implemented in Molecular Evolutionary Genetics Analysis (MEGA X) (Kumar et al., 2018). Alignments were manually inspected, and conserved blocks for phylogenetic analysis were selected with GBLOCKS (Castresana, 2000). Phylogenetic analysis was performed based on two methods: the Bayesian inference in MrBayes 3.2 (Ronquist et al., 2012) and Maximum Likelihood (ML) in IQ-Tree 2.1.3 (Minh et al., 2020). The posterior probability and the bootstrap test were presented as statistical supports for the internal nodes for Bayesian and ML trees, respectively. To select the best-fit models of amino acid substitution based on BIC and AIC scores, we used ModelTest-NG 0.1.5 (Darriba et al., 2020), available on the CIPRES Science Gateway v.3.3 (Miller et al., 2010). We performed two independent runs, each with four chains of 12, 000, 000 generations of Markov chain Monte Carlo (MCMC) algorithms for amino acid sequences. The first 25% of generations were deleted as burn-in. Tracer 1.7.1 (Rambaut et al., 2018) was then used to verify data obtained by the convergence of Markov chains and satisfactory effective sample sizes (>200). Trees were visualized and edited in FigTree v.1.4.4 (http://tree.bio.ed.ac.uk/). We used TimeTree (Hedges et al., 2006) and constructed a simplified species tree using the divergence time between pairs of representative organisms from each major group.

*Gene structure*

Intron/exon organization in *PSY* genes was analyzed to better understand the rules governing gene structure and their consequences on protein function and evolutionary patterns among species (Wang et al., 2014). Specifically, a comparative analysis was conducted using genomic sequences and CDS for *PSY* from 13 representative species (*Physcomitrella patens*, *Selaginella moellendorffii*, *Brachypodium distachyon*, *Setaria italica*, *Oryza sativa*, *Sorghum bicolor*, *Solanum lycopersicum*, *Solanum tuberosum*, *Eucalyptus grandis*, *Citrus sinensis*, *Arabidopsis thaliana*, *Brassica rapa*, and *Glycine max*). The Gene Structure Display Server (GSDS 2.0) (Hu et al., 2015) was used to display intron/exon organization and intron phase patterns, along with a phylogenetic tree for the representative species constructed using protein sequence alignments of those species and following methods described in the previous section.

*Identification of transmembrane domains and conserved motifs*

The presence of transmembrane domains in PSY protein sequences was predicted in TMHMM-2.0 (Krogh et al., 2001), provided by CBS Prediction Servers and PROTTER (Omasits et al., 2014). Potential functional motifs were identified using the MEME utility program (Multiple Expectation Maximization for Motif Elicitation) (Bailey et al., 2006). The sequence logo was constructed in WebLogo (Crooks et al., 2004).

### Gene expression analysis

To determine tissue specificity and intensity of *PSY* gene expression in *A*. *thaliana*, *G*. *max*, *O*. *sativa*, and *S*. *bicolor*, we used microarray and RNA-seq data from the GENEVESTIGATOR website (Hruz et al., 2008), along with its hierarchical clustering tool. The highest expression values were considered for genes with more than one probe set. Expression data were normalized and hierarchically clustered based on Pearson coefficients. The potential for *PSY* gene expression in different anatomical regions and developmental stages is represented with heat maps.

## Results

### Identification of PSY homologs

After analyzing 351 *PSY* gene sequences from 166 species (Table S1), we found that red algae, brown algae, fungi, and arthropods have only one *PSY* gene. This pattern is generally true for the prokaryotes, algae, and cyanobacteria species. Most plant species have more than one *PSY* gene, but the following only have one: bryophytes *Marchantia polymorpha* and *Sphagnum fallax*; lycophyte *Selaginella moellendorffii*; monocots *Zostera marina* and *Saccharum spontaneum*; Brassicaceae species *Arabidopsis halleri*, *A*. *lyrata*, *A*. *thaliana*, *Boechera stricta*, *Capsella grandiflora*, and *C*. *rubella*; as well as eudicots *Carica papaya* and *Beta vulgaris*. 

### Phylogenetic analysis

**Figure 1 -:**
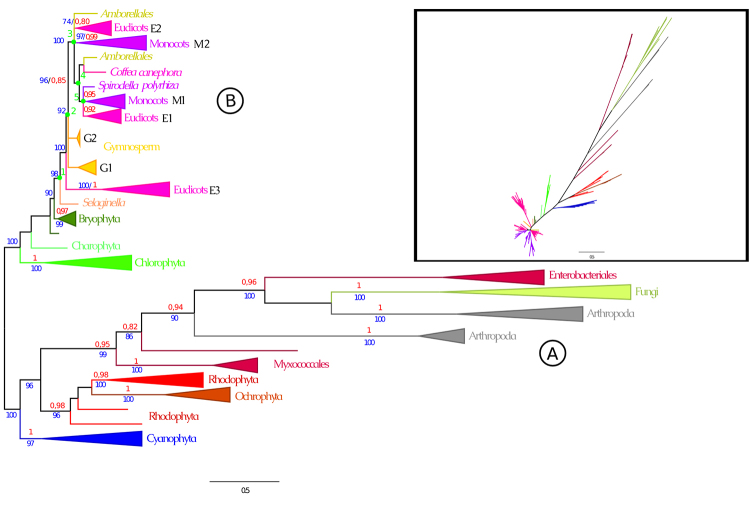
Phylogenetic tree for *PSY* genes in representative Viridiplantae, fungi, arthropods, brown algae, red algae, cyanobacteria, and prokaryote lineages, providing insight into PSY origin and diversification among terrestrial plants. Figures show the resulting tree of the Bayesian inference analysis and present both posterior probabilities (numbers in red) with a cut-off point of 0, 8 and bootstrap values (numbers in blue) with a cut-off point of 70. The tree was rooted with prokaryotes as a sister group of the other organisms (root selected manually in FigTree v.1.4.4). Bayesian and maximum likelihood analyses were performed using PSY amino acid sequences from 166 selected representative species. Five main nodes that potentially mark taxonomic divergence during plant evolution (Nodes 1, 2, 3, 4 and 5) are marked with green circles. Inside the square in the upper right corner of the figure is the original unrooted tree.

To understand PSY phylogeny and diversification patterns, we inferred a phylogenetic tree with 351 PSY amino acid sequences spanning 166 species. The dataset includes 20 sequences from bacteria, 13 from red algae, 11from brown algae, 13 from cyanobacteria, eight from algae, five from bryophytes, 10 from fungi, seven from arthropods, one from charophytes, one from lycophytes, four from gymnosperms, two from early angiosperms, and 256 from angiosperms (monocotyledons and eudicotyledons) (Table S1). Alignments used for the phylogenetic analysis consisted of 264 sites. The best model suggested for the protein dataset, for both BIC and AIC scores, was LG+I+G4. The tree topology presented in Figure 1 results from the Bayesian inference analysis and shows both posterior probability and bootstrap values.

We identified two major groups (A and B) in the phylogenetic tree constructed using all 351 sequences (Figure 1). Identified as group A are brown algae Ochrophyta, red algae Rhodophyta, blue algae Cyanophyta, fungi, bacteria, and arthropods. Group B included angiosperms (eudicots subgroups E1, E2 and E3, and monocots subgroups M1 and M2), gymnosperms (subgroups G1 and G2), the early angiosperm *Amborella trichopoda*, sequences of bryophytes, lycophyte *Selaginella moellendorffii*, green algae chlorophyta and the charophyta *Klebsormidium nitens*. Terrestrial plants had sequences that formed distinct groups in the phylogeny. Interestingly, PSY3 gene orthologs (subgroup E3), identified and functionally characterized in tomatoes, includes only eudicotyledon species and seems to be the most divergent one, being located at the more external position than other angiosperms and even gymnosperms.

### Patterns of gene duplication and gene loss

After analyzing the angiosperm group in the phylogenetic tree, we observed that most eudicots had at least one representative species in subgroup E3 (Figure S3), except for Brassicaceae family and *Lupinus angustifolius*, *Beta vulgaris*, *Solanum tuberosum* species. The absence of members from a given PSY group in each species might represent gene loss. Still, it could also result from an incomplete or locally misassembled genome, improper annotation, or failure to meet our screening criteria. In subgroup E3, *Glycine max* presented two PSY members that were grouped together. Because other Fabaceae family members had only one gene in this cluster, the two grouped genes probably resulted from a WGD (whole genome duplication) event. *Mimulus guttatus*, *Linum usitatissimum*, and *Kalanchoe laxiflora* species in subgroup E3 also may have experienced WGD events, with two gene members clustered together. In this subgroup, two branches with *Citrus clementina* and *Citrus sinensis* were clustered in a single branch, suggesting duplication in the *Citrus* base genus. 

Subgroups E1, E2, M1, M2, and two sequences from early angiosperm *Amborella trichopoda* were positioned in the same branch (Figure 1). The subgroups E1 and E2 (Figures S4 and S5 respectively) grouped eudicot sequences, while the subgroups M1 and M2 (Figures S6 and S7, respectively) grouped monocot sequences. Based on these results, we suggest that the duplication events leading to the emergence of these groups occurred prior to the monocot-eudicot divergence. In subgroup E1 (Figure S4), several recent duplications appear to have occurred, with one duplication in *Glycine max*, two duplications in *Linum usitatissimum* that are probably separate WGD events, one duplication in *Malus domestica*, and one duplication in *Gossypium raimondii*. Additionally, in this clade, two branches with the species *Kalanchoe laxiflora* and *Kalanchoe fedtschenko* were clustered in a single branch, suggesting duplication in the *Kalanchoe* base genus. *Salix purpurea* also had two subsequent duplications, each containing a sequence from *Populus trichocarpa*, suggesting duplication at the base of the Salicaceae family. In subgroupM1 (Figure S6), four monocots had duplications suggestive of WGD: *Triticum turgidum*, *Zea mays*, *Eragrostis tef*, and *Musa acuminata*. In subgroup E2 (Figure S5), species with duplications suggestive of WGD were *Glycine max*, *Solanum tuberosum*, *Actinidia chinensis*, *Eucalyptus grandis*, *Citrus clementina*, *Eutrema salsugineum*, and *Malus domestica*. In this subgroup two branches with the species *Cynara cardunculus* and *Helianthus annuus* were clustered in a single branch, suggesting duplication at the Asteraceae base Family. In subgroup M2 (Figure S7), species that may have experienced WGD were *Musa acuminata*, *Leersia perrieri*, *Eragrostis tef*, *Panicum virgatum*, and *Zea mays*.

### Comparative analysis of gene structure and conserved domains and motifs in PSY proteins

**Figure 2 -:**
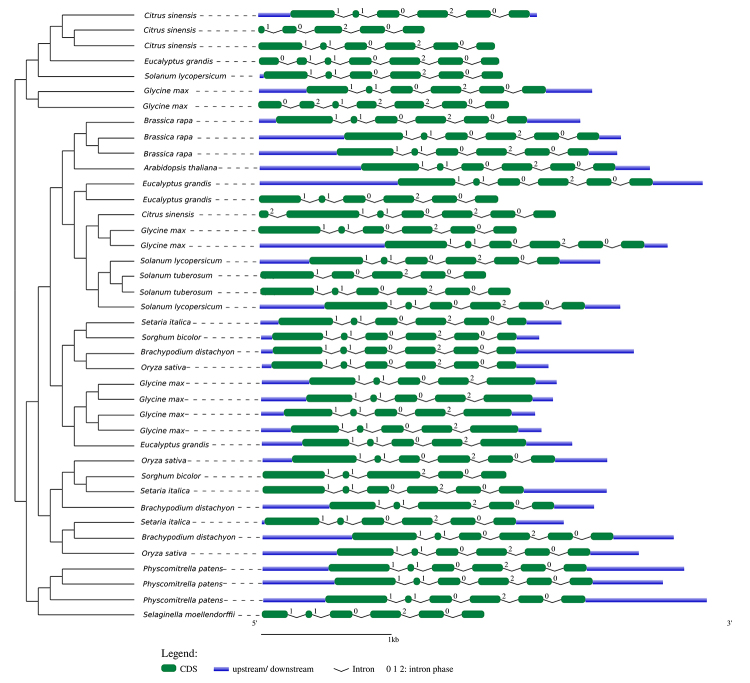
Exon-intron organization of *PSY* genes in terrestrial plants. PSY sequences from 13 representative species (*Physcomitrella patens*, *Selaginella moellendorffii*, *Brachypodium distachyon*, *Setaria italica*, *Oryza sativa*, *Sorghum bicolor*, *Solanum lycopersicum*, *Solanum tuberosum*, *Eucalyptus grandis*, *Citrus sinensis*, *Arabidopsis thaliana*, *Brassica rapa*, *Glycine max*) are presented. Gene features are displayed on a Bayesian phylogenetic tree.

**Figure 3-:**
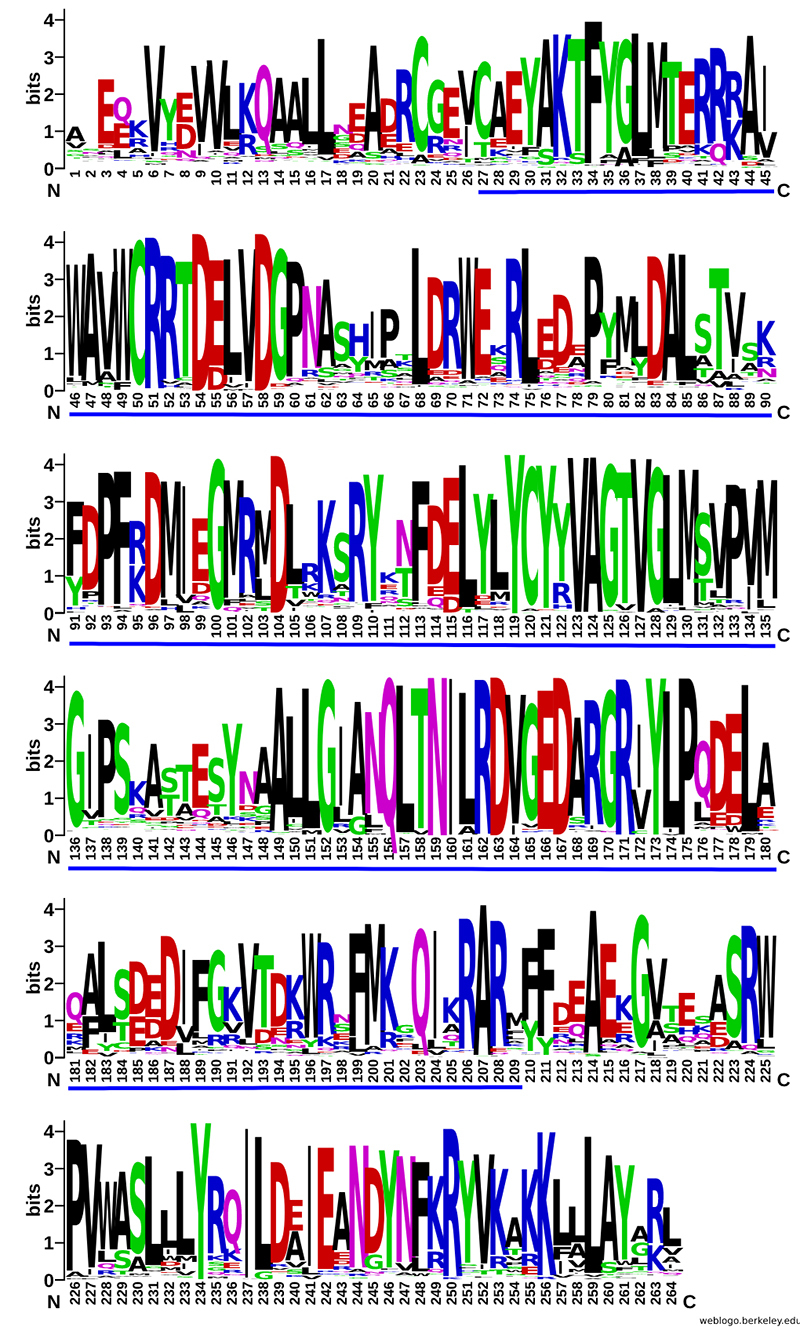
Amino acid sequence logo of PSY alignments from angiosperm species, highlighting the high gene conservation. The vertical axis indicates information content of a sequence position, in bits (log2 4 = 2 bits for DNA/RNA, log2 20 = 4.3 bits for protein). The height of the y-axis is the maximum entropy for a given sequence type. The horizontal axis indicates the residue number. The blue line indicates the SQS_PSY domain.

Exon and intron length (in base pairs) was manually counted via aligning cDNA sequences with their corresponding genomic DNA sequences. Analysis of gene structure for exon-intron organization revealed that the number of introns per gene varied from four to five, with a few exceptions. Intron number and gene organization were fairly conserved among species (Figure 2). The length of PSY amino acid sequences ranged from 300 to 440. To analyze functional motifs, we searched for conserved domains in representative proteins from the retrieved sequences. Examining amino acid sequences encoded by these genes allowed us to identify conserved sites and motifs characteristic of the PSY family. We inferred that proteins encoded by *PSY* genes are highly conserved and feature a common domain, the SQS-PSY (squalene/ phytoene synthase-Pfam accession no. 00494) domain (Figure 3). Predictions of transmembrane structures revealed that transmembrane sequences were absent in *PSY* genes, suggesting that PSY are soluble proteins and not associated with membrane systems.

### Gene expression analysis of PSY in monocot and eudicot model species

We used RNA-seq and microarray data from GENEVESTIGATOR to analyze the global expression profile of *PSY* genes from four model species representing monocot (*Oryza sativa* and *Sorghum bicolor*) and eudicot (*Arabidopsis thaliana* and *Glycine max*) groups. We analyzed 127 anatomical parts and 10 developmental stages from *A*. *thaliana*, 28 anatomical parts and eight developmental stages from *G*. *max*, nine anatomical parts and five developmental stages from *S*. *bicolor*, and 42 anatomical parts and nine developmental stages from *O*. *sativa*. In each species, *PSY* was ubiquitously expressed across all developmental stages and anatomical parts, with species-specific differences in the tissue and stage that had higher expression (Table 1, Figures S8-15). *Arabidopsis thaliana* has only one *PSY* gene (*AthPSY1*) in its genome, included in Subgroup E2 and highly expressed in sperm cells, mesophyll protoplasts, seedling cultures, shoots, leaves, and inflorescence parts. *AthPSY1* expression was high at all developmental stages and decreased during final ripening (Table 1, Figure S8-9). Additionally, *AthPSY1* expression was low in roots but upregulated in perturbation experiments. *Glycine max* is a model oleaginous crop and has eight *PSY* paralog genes: Glyma.14G209700 and Glyma.02G240200 in subgroup E2; Glyma.14G031200, Glyma.02G283400, Glyma.18G111900, and Glyma.08G306200 in subgroup E1; Glyma.18G000600 and Glyma.11G256400 in subgroup E3. *GmaPSY* genes exhibited medium to high expression in developmental stages and anatomical parts (Table 1, Figure S10-11). Glyma.18G000600 was lowly expressed under normal conditions but upregulated in perturbation experiments with biotic stress (e.g., fungi, insect pests, and parasites). Glyma.11G256400 was not detected in either RNA-seq or microarray data. Hierarchical clustering revealed that *GmaPSY* genes were grouped together on the phylogenetic tree. *Oryza sativa* has three PSY genes that are highly expressed across all developmental stages, especially during heading (Table 1, Figure S12-13). The two *PSY* genes from *S*. *Bicolor* presented a similar expression pattern, with medium to high expression detected in all tissues and developmental stages (Figure S14-15).

**Table 1 -:** Gene expression analysis from Genevestigator showing expression pattern of PSY in anatomical parts and developmental stages of *Arabidopsis thaliana*, *Glycine max*, *Oryza sativa and Sorghum bicolor*.

Species	Accesion number	Location on phylogeny	Expression data		Source
			Development stadios	Anatomical parts	
*Arabidopsis thaliana*	AT5G17230.1	Subgroup E2	The expression was high in all stages, except in final ripening	The expression detected in almost all anatomical parts, with high expression level detected in sperm cell, mesophyll cell protoplast, root culture, seedling culture, shoot cell and tissues, leaf cell and tissues, inflorescence parts	Figure S8 and S9
*Glycine max*	Glyma.14G209700	Subgroup E2	The expression was detected in all stages, with higher expression level in main shoot growth, stem elongation, inflorescence formation and flowering	Higher expression was detected in cotyledon, shoot apex, hypocotyl, flower, pod, pericarp (pod wall), shoot, stem internode, leaf, unifoliolate leaf, trifoliolate leaf, shoot apex, shoot apical meristem	Figure S10 and S11
	Glyma.02G240200	Subgroup E2	The expression was detected in all stages, with higher expression level in main shoot growth, stem elongation, inflorescence formation and flowering	Higher expression was detected in cotyledon, shoot apex, hypocotyl, flower, pod, pericarp (pod wall), shoot, stem internode, leaf, unifoliolate leaf, trifoliolate leaf, shoot apex, shoot apical meristem	Figure S10 and S11
	Glyma.14G031200	Subgroup E1	The expression was detected in all stages, with higher expression level in main shoot growth and inflorescence formation	Higher expression was detected in shoot apex, flower, pod, shoot, leaf, unifoliolate leaf, trifoliolate leaf shoot apex, shoot apical meristem	Figure S10 and S11
	Glyma.02G283400	Subgroup E1	The expression was detected in all stages, with higher expression level in main shoot growth and inflorescence formation	Higher expression was detected in shoot apex, flower, pod, shoot, leaf, unifoliolate leaf, trifoliolate leaf shoot apex, shoot apical meristem	Figure S10 and S11
	Glyma.18G111900	Subgroup E1	The expression was detected in all stages, with higher expression level in main shoot growth, inflorescence formation and flowering	Higher expression was detected in cotyledon, shoot apex, flower, pod, shoot, leaf, unifoliolate leaf, trifoliolate leaf, shoot apex, shoot apical meristem	Figure S10 and S11
	Glyma.08G306200	Subgroup E1	The expression was detected in all stages, with higher expression level in main shoot growth, inflorescence formation and flowering	Higher expression was detected in cotyledon, shoot apex, flower, pod, shoot, leaf, unifoliolate leaf, trifoliolate leaf, shoot apex, shoot apical meristem	Figure S10 and S11
	Glyma.18G000600	Subgroup E3	Low level of expression in all stages	Low expression was detected in root	Figure S10 and S11
*Oryza sativa*	LOC_Os09g38320	Subgroup M2	High level of expression in all stages	Higher expression was detected in seedling, coleoptile, leaf, root, radicle, panicle, spikelet, floret, stigma, ovary, panicle branch, peduncle, leaf, blade (lamina), central vein, collar	Figure S12 and S13
	LOC_Os06g51290	Subgroup M2	High level of expression in all stages	Higher expression was detected in seedling, leaf, panicle, spikelet, floret, ovary, panicle branch, shoot, leaf, blade (lamina), central vein	Figure S12 and S13
	LOC_Os12g43130	Subgroup M1	High level of expression in all stages	Higher expression was detected in shoot, leaf, blade (lamina), central vein, flag leaf	Figure S12 and S13
*Sorghum bicolor*	Sobic.002G292600	Subgroup M2	The expression was detected in all stages, with higher expression level in flowering stage	Medium expression was detected in all 9 anatomical parts with higher expression in leaf	Figure S14 and S15
	Sobic.008G180800	Subgroup M1	The expression was detected in all stages, with higher expression level in stem elongation stage	Medium expression was detected in all 9 anatomical parts	Figure S14 and S15

## Discussion

The *PSY* gene family encodes a rate-limiting enzyme in carotenoid biosynthesis and is ubiquitous in plants (Welsch et al., 2000; Yang et al., 2005). The first member of the angiosperm PSY gene (PSY1) was cloned from tomato fruits, and its expression correlated with lycopene accumulation (Bartley et al., 1992; Ray et al., 1992). Since then, many researchers have identified and characterized PSY family members in a variety of plants, demonstrating their importance in controlling carotenoid biosynthesis and their association with pigment diversity and stress response (Shao et al., 2018). While most plant species have two or more *PSY* paralog genes, some have only one (e.g., *A*. *thaliana*). The presence of multiple paralogs could potentially explain carotenogenesis in various tissues (Fantini et al., 2013; Wang et al., 2014; Yuan et al., 2015), given that the expression profiles of different PSY isoforms exhibited tissue specificity. For example, in tomato (*Solanum lycopersicum*), PSY1 is expressed in the fruit at levels that correlate with carotenoid content, while PSY2 is expressed in leaves; PSY3 expression is specific to roots and also conditional on being under stress (Nisar et al., 2015). These findings spur new questions regarding such an important gene family from an evolutionary point of view. Thus, in this study, we used a phylogenetic approach to gain insights into the evolution and diversification of the PSY gene family. After searching available whole-genome sequences of plants, algae, red algae, brown algae, fungi, bacteria, cyanobacteria, and arthropods in GenBank, we identified 351 PSY genes across 166 species. We confirmed that most species have more than one PSY gene and that most duplications occurred after angiosperm diversification since angiosperms have the most significant number of genes.

In this study, we observed a discrepancy between the gene (Figure 1) and species (Figure S1) tree. Fungi and arthropod PSY sequences were grouped with prokaryote PSY, and ochrophyta PSY grouped with rodophyta PSY, suggesting the occurrence of horizontal gene transfer. Horizontal gene transfer is the acquisition of genes from organisms other than a direct ancestor (Crisp et al., 2015). Our results corroborate with other studies, such those that have demonstrated that the ochrophytahas a red alga-derived plastid through eukaryote-eukaryote endosymbiosis (Ševčíková et al., 2015; Sibbald and Archibald, 2020; Azuma et al., 2022). Bacteria can obtain genes from other species via horizontal gene transfer, resulting in the genes being distributed among different species. Previous studies have shown that the horizontal transfer of carotenoid biosynthesis genes plays a major role in the distribution of carotenoid pathways across unrelated phylogenetic lineages (Phadwal, 2005; Klassen, 2010). The lateral transfer has been reported for some arthropod species (red aphids, spider mites, and gall midges) that received enzymatic machinery for carotenoid biosynthesis from fungi (Moran and Jarvik, 2010; Grbić et al., 2011; Cobbs et al., 2013). In prokaryotes, horizontal gene transfer has great adaptive significance, although its impact on eukaryotic evolution remains unclear. Some evidence suggests that the presence of certain genes in various plant-feeding insects, mites, and fungi can only be explained by horizontal gene transfer (Moran and Jarvik, 2010; Novikova et al., 2010; Walsh et al., 2013; Wybouw et al., 2016). 

We identified five main nodes in the angiosperms subgroups PSY gene tree (Figure 1) that potentially mark taxonomic divergence during plant evolution. Node 1 probably corresponds to the divergence between lycophytes and other plants (gymnosperms + angiosperms). After lycophyte diversification, the PSY gene was duplicated in the spermatophyte ancestor. However, the ancestor paralog was maintained only in the eudicot lineage, which gave rise to subgroup E3. Node 2 appears to mark the divergence between gymnosperms and angiosperms. Gymnosperms inherited two copies, whereas angiosperms inherited only one copy. Node 3 presents the duplication events of *PSY* in the angiosperm clade. One copy remained in the three lineages from Node 4 (monocots, eudicots, and *Amborella*). The other paralogs were acquiredin eudicots (subgroup E2), monocots (subgroup M2), and *Amborella*. Node 4 is split into two branches. The first groupis a *Coffea* species with *Amborella*, suggesting a horizontal transfer event. The second group leads to Node 5, which marks the separation between monocots and eudicots. Figure S2 summarizes these patterns of gene duplication and loss.

Using RNA-seq and microarray data, we demonstrated that genes Glyma.18G000600 and Glyma.11G256400 in subgroup E3 were expressed when exposed to biotic stress. This characteristic resembles tomato PSY3, which is expressed during fungal colonization. In subgroup M2, the genes Sobic.002G292600 (*S*. *bicolor*) and LOC_Os9g38320 (*O*. *sativa*) exhibit abiotic-stress-inducible expression, similar to PSY3 in Poaceae (Dibari et al., 2012). The absence of subgroup E3 PSY in some species, such as those from Brassicaceae, suggests that this paralog was lost in the ancestor of the family.

Our results show a duplication pattern consistent with WGD, indicating that such events may be the main source of PSY duplication. Our findings corroborate with previous studies that have found evidence of WGD events in *Salix purpurea* and *Populus trichocarpa* (Tuskan et al., 2006; Koenen et al., 2021). Fabaceae experienced three WGD events, one in the ancestor of the family and the other two occurring independently in subfamilies Detarioideae and Papilionoideae (Koenen *et al*., 2021). Polyploidy in grasses is an ongoing process (Levy and Feldman, 2002), further supporting the possibility of WGD.

Our study inferred the phylogenetic tree of the PSY gene family in various species and contributed to the knowledge about the evolutionary history of this gene family. The divergence between subgroup E3 and the other subgroups of plants most likely occurred after an ancient replication, when other terrestrial plants besides eudicots lost the subgroup E3 copy over time. Mapping the gain and losses of PSY genes in the phylogenetic tree, we got insights into the process leading to the diversification of this gene family. It is well known that gene gain and loss are significant forces driving evolution (Delabre et al., 2020). Thus, the preponderance of PSY duplicates in plant genomes could explain the capacity to evolve different carotenoids profiles associated with ecological circumstances, such as biotic stress.

## Supplementary material

The following online material is available for this article:

Table S1 - Detailed data of retrieved sequences and species used in this study.

Figure S1 - Simplified species tree using the divergence time between pairs of representative organisms used from each major group.

Figure S2 - Scheme of *PSY* gene duplications and losses.

Figure S3 - Phylogenetic relationships among *PSY* genes belonging to subgroup E3 from Figure 1.

Figure S4 - Phylogenetic relationships among PSY genes belonging to Subgroup E1 from Figure 1.

Figure S5 - Phylogenetic relationships among PSY genes belonging to Subgroup E2 from Figure 1.

Figure S6 - Phylogenetic relationships among PSY genes belonging to subgroup M1 from Figure 1.

Figure S7 - Phylogenetic relationships among PSY genes belonging to Subgroup M2 from Figure 1.

Figure S8 - Gene expression analysis across developmental stages of *Arabidopsis thaliana* performed using GENEVESTIGATOR database.

Figure S9 - Gene expression analysis across anatomical parts of *Arabidopsis thaliana* performed using GENEVESTIGATOR.

Figure S10 - Gene expression analysis across anatomical parts of *Glycine max* performed using GENEVESTIGATOR.

Figure S11 - Gene expression analysis across developmental stages of *Glycine max* performed using GENEVESTIGATOR.

Figure S12 - Gene expression analysis across developmental stages of *Oryza sativa* performed using GENEVESTIGATOR.

Figure S13 - Gene expression analysis across anatomical parts of *Oryza sativa* performed using GENEVESTIGATOR.

Figure S14 - Gene expression analysis across developmental stages of *Sorgum bicolor* performed using GENEVESTIGATOR.

Figure S15 - Gene expression analysis across anatomical parts of *Sorgum bicolor* performed using GENEVESTIGATOR.
