# Supplementary material for: Molecular evolution and diversification of phytoene synthase (PSY) gene family
Source: Genet Mol Biol. 2022 Dec 19;45(4):e20210411. doi: 10.1590/1678-4685-GMB-2021-0411 (PMC9764326; doi:10.1590/1678-4685-GMB-2021-0411)
Supplement: Table S1 - [file 1415-4757-GMB-45-4-e20210411-s1.pdf]

*Genetics and Molecular Biology*

https://doi.org/10.1590/1678-4685-GMB-2021-0411

# Supplementary material to "Molecular evolution and diversification of phytoene synthase (PSY) gene family"

**Table S1** - Detailed data of retrieved sequences and species used in this study.

| Species | Acronymon | Score | Evalue | Transcript Name | CDS Acession Number | Location | Classification group | Order | Query |
|---|---|---|---|---|---|---|---|---|---|
| *Anaeromyxobacter sp* | *Asp* | *165* | *2,70E-39* | *ABS28258* | *ABS28258* | *Chromosome: 4,772,703-4,773,635 reverse strand* | *Prokariotae* | *Myxoccocales* | *Solyc03g031860.2.1* |
| *Candidatus Parcubacteria bacterium* | *Cpb* | *356* | *3,00E-118* | *MBC7822343* | *MBC7822343.1* | | *Prokariotae* | *Candidatus parcubacteria* | *Solyc03g031860.2.1* |
| *Cronobacter turicensis* | *Ctu* | *333* | *2,00E-86* | *FN543093* | *FN543093.2* | | *Prokariotae* | *Enterobacteriales* | *Pantoeae ananatis D90087.2* |
| *Cronobacter universalis* | *Cun* | *333* | *2,00E-86* | *CP012257* | *CP012257.1* | | *Prokariotae* | *Enterobacteriales* | *Pantoeae ananatis D90087.2* |
| *Erwinia gerundensis* | *Ege* | *129* | *6,90E-32* | *CUU22454* | *CUU22454* | *Chromosome 1: 233,386-234,312 reverse strand.* | *Prokariotae* | *Enterobacteriales* | *Solyc03g031860.2.1* |
| *Erwinia sp.* | *Esp* | *162* | *6,70E-33* | *RRZ89314* | *RRZ89314* | *NODE_11_length_177429_cov_45.8436 17769 to 18209 (-)* | *Prokariotae* | *Enterobacteriales* | *Solyc03g031860.2.1* |
| *Mixta calida* | *Mca* | *345* | *3,00E-90* | *CP061511* | *CP061511.1* | | *Prokariotae* | *Enterobacteriales* | *Pantoeae ananatis D90087.2* |
| *Mixta gaviniae* | *Mga* | *353* | *2,00E-92* | *CP026377* | *CP026377.1* | | *Prokariotae* | *Enterobacteriales* | *Pantoeae ananatis D90087.2* |

| Species | Acronymon | Score | Evalue | Transcript Name | CDS Acession Number | Location | Classification group | Order | Query |
|---|---|---|---|---|---|---|---|---|---|
| *Myxococcus fulvus* | *Mfu* | *199* | *3,30E-31* | *SET01046* | *SET01046* | *SuperContig Ga0131203_101: 961,439-962,455 forward strand.* | *Prokariotae* | *Mixococcales* | *Solyc03g031860.2.1* |
| *Myxococcus hansupus* | *Mha* | *205* | *9,60E-50* | *AKQ69173* | *AKQ69173* | *Chromosome: 7,548,393-7,549,391 reverse strand.* | *Prokariotae* | *Mixococcales* | *Solyc03g031860.2.1* |
| *Myxococcus macrosporus* | *Mma* | *206* | *1,50E-50* | *ATB45335* | *ATB45335* | *Chromosome: 1,082,269-1,083,282 forward strand.* | *Prokariotae* | *Mixococcales* | *Solyc03g031860.2.1* |
| *Myxococcus stipitatus* | *Msi* | *204* | *2,50E-50* | *AGC42255* | *AGC42255* | *Chromosome: 1,121,269-1,122,288 forward strand.* | *Prokariotae* | *Mixococcales* | *Solyc03g031860.2.1* |
| *Myxococcus virescens* | *Mvi* | *201* | *3,90E-50* | *SDD40313* | *SDD40313* | *SuperContig Ga0070493_101: 905,667-906,677 reverse strand.* | *Prokariotae* | *Mixococcales* | *Solyc03g031860.2.1* |
| *Myxococcus xanthus* | *Mxa* | *202* | *5,10E-52* | *ABF86184* | *ABF86184* | *Chromosome: 1,021,919-1,022,929 forward strand* | *Prokariotae* | *Mixococcales* | *Solyc03g031860.2.1* |
| *Pantoea agglomerans* | *Pag* | *669* | *0.0* | *CP031650* | *CP031650.1* | | *Prokariotae* | *Enterobacteriales* | *Pantoeae ananatis D90087.2* |
| *Pantoea ananatis* | *Pan* | *1678* | *0.0* | *D90087* | *D90087.2* | | *Prokariotae* | *Enterobacteriales* | *Pantoeae ananatis D90087.2* |
| *Pantoea ananatis* | *Pan* | *1645* | *0.0* | *CP001875* | *CP001875.2* | | *Prokariotae* | *Enterobacteriales* | *Pantoeae ananatis D90087.2* |
| *Pantoea eucalypti* | *Peu* | *625* | *2,00E-174* | *CP045721* | *CP045721.1* | | *Prokariotae* | *Enterobacteriales* | *Pantoeae ananatis D90087.2* |
| *Pantoea stewartii* | *Pst* | *883* | *0.0* | *CP017591* | *CP017591.1* | | *Prokariotae* | *Enterobacteriales* | *Pantoeae ananatis D90087.2* |
| *Pantoea vagans* | *Pva* | *662* | *0.0* | *KT156635* | *KT156635.1* | | *Prokariotae* | *Enterobacteriales* | *Pantoeae ananatis D90087.2* |
| *Ahnfeltiopsis flabelliformis* | *Afl* | *312* | *2,00E-102* | *ATQ35976* | *ATQ35976* | | *Rhodophyta* | *Gigartinales* | *Solyc03g031860.2.1* |
| *Betaphycus gelatinus* | *Bge* | *300* | *3,00E-98* | *ATQ35975* | *ATQ35975* | | *Rhodophyta* | *Gigartinales* | *Solyc03g031860.2.1* |

| Species | Acronymon | Score | Evalue | Transcript Name | CDS Acession Number | Location | Classification group | Order | Query |
|---|---|---|---|---|---|---|---|---|---|
| *Chondrus crispus* | *Cci* | *291* | *1,00E-95* | *ATQ35984* | *ATQ35984* | | *Rhodophyta* | *Gigartinales* | *Solyc03g031860.2.1* |
| *Cyanidioschyzon merolae* | *Cme* | *317* | *4,00E-103* | *XP_005537057* | *XP_005537057* | | *Rhodophyta* | *Cyanidiales* | *Solyc03g031860.2.1* |
| *Dumontia simplex* | *Dsi* | *306* | *3,00E-100* | *ATQ35978* | *ATQ35978* | | *Rhodophyta* | *Gigartinales* | *Solyc03g031860.2.1* |
| *Galdieria sulphuraria* | *Gsu* | *498* | *1,70E-91* | *EME29253* | *EME29253* | *SuperContig scaf_26: 15,896-17,400 reverse strand* | *Rhodophyta* | *Cyanidiales* | *Solyc03g031860.2.1* |
| *Gracilaria chouae* | *Gco* | *295* | *1,00E-95* | *ATQ35977* | *ATQ35977* | | *Rhodophyta* | *Gracilariales* | *Solyc03g031860.2.1* |
| *Gracilariopsis chorda* | *Gch* | *305* | *5,00E-100* | *PXF46860* | *PXF46860* | | *Rhodophyta* | *Gracilariales* | *Solyc03g031860.2.1* |
| *Gracilariopsis lemaneiformis* | *Gle* | *305* | *1,00E-99* | *ATQ35980* | *ATQ35980* | | *Rhodophyta* | *Gracilariales* | *Solyc03g031860.2.1* |
| *Grateloupia catenata* | *Gca* | *293* | *5,00E-95* | *ATQ35982* | *ATQ35982* | | *Rhodophyta* | *Halymeniales* | *Solyc03g031860.2.1* |
| *Grateloupia livida* | *Gli* | *297* | *1,00E-96* | *ATQ35979* | *ATQ35979* | | *Rhodophyta* | *Halymeniales* | *Solyc03g031860.2.1* |
| *Grateloupia turuturu* | *Gtu* | *298* | *7,00E-97* | *ATQ35985* | *ATQ35985* | | *Rhodophyta* | *Halymeniales* | *Solyc03g031860.2.1* |
| *Porphyridium purpureum* | *Ppu* | *306* | *8,00E-99* | *KAA8491175* | *KAA8491175* | | *Rhodophyta* | *Porphyridiales* | *Solyc03g031860.2.1* |
| *Desmarestia viridis* | *Dvi* | *290* | *6,00E-94* | *ATQ35993* | *ATQ35993* | | *Ochrophyta* | *Desmarestiales* | *Solyc03g031860.2.1* |
| *Dictyopteris undulata* | *Dun* | *286* | *1,00E-92* | *ATQ35997* | *ATQ35997* | | *Ochrophyta* | *Dictyotales* | *Solyc03g031860.2.1* |
| *Ectocarpus siliculosus* | *Esi* | *294* | *1,00E-94* | *CBJ31337* | *CBJ31337* | *scaffold sctg_244* | *Ochrophyta* | *Ectocarpales* | *Solyc03g031860.2.1* |
| *Ishige okamurae* | *Iok* | *293* | *6,00E-95* | *ATQ35992* | *ATQ35992* | | *Ochrophyta* | *Ishigeales* | *Solyc03g031860.2.1* |
| *Punctaria latifolia* | *Pla* | *295* | *3,00E-95* | *ATQ35994* | *ATQ35994* | | *Ochrophyta* | *Ectocarpales* | *Solyc03g031860.2.1* |
| *Sargassum hemiphyllum* | *She* | *295* | *1,00E-96* | *ATQ36002* | *ATQ36002* | | *Ochrophyta* | *Fucales* | *Solyc03g031860.2.1* |
| *Sargassum henslowianum* | *Shn* | *295* | *2,00E-96* | *ATQ36001* | *ATQ36001* | | *Ochrophyta* | *Fucales* | *Solyc03g031860.2.1* |

| Species | Acronymon | Score | Evalue | Transcript Name | CDS Acession Number | Location | Classification group | Order | Query |
|---|---|---|---|---|---|---|---|---|---|
| *Sargassum thunbergii* | *Sth* | *297* | *3,00E-96* | *ATQ35998* | *ATQ35998* | | *Ochrophyta* | *Fucales* | *Solyc03g031860.2.1* |
| *Sargassum vachellianum* | *Sva* | *295* | *1,00E-95* | *ATQ36000* | *ATQ36000* | | *Ochrophyta* | *Fucales* | *Solyc03g031860.2.1* |
| *Scytosiphon lomentaria* | *Slo* | *295* | *2,00E-95* | *ATQ35995* | *ATQ35995* | | *Ochrophyta* | *Ectocarpales* | *Solyc03g031860.2.1* |
| *Undaria pinnatifida* | *Upi* | *291* | *4,00E-94* | *ATQ35996* | *ATQ35996* | | *Ochrophyta* | *Laminariales* | *Solyc03g031860.2.1* |
| *Cyanobacteria bacterium* | *Cba* | *358* | *3,00E-121* | *DNME01000182.1* | *HBB31740.1* | | *Cyanophyta* | *Chroococcales* | *Solyc03g031860.2.1* |
| *Cyanobacteria bacterium* | *Cba* | *357* | *1,00E-120* | *RFIF01000104.1* | *RMF69129.1* | | *Cyanophyta* | *Chroococcales* | *Solyc03g031860.2.1* |
| *Cyanothece sp.* | *Csp* | *360* | *8,00E-120* | *NET36663* | *NET36663* | *JAAHFT010000438.1:6921-7850* | *Cyanophyta* | *Oscillatoriales* | *Solyc03g031860.2.1* |
| *Desertifilum sp.* | *Dsp* | *365* | *7,00E-122* | *NES97327* | *NES97327* | | *Cyanophyta* | *Oscillatoriales* | *Solyc03g031860.2.1* |
| *Elainella sp.* | *Els* | *348* | *3,00E-115* | *MBF2049978* | *MBF2049978* | *JACYLO010000073.1:11222-12151* | *Cyanophyta* | *Synechococcales* | *Solyc03g031860.2.1* |
| *Hydrocoleum sp.* | *Hsp* | *348* | *5,00E-115* | *OZH53043* | *OZH53043* | *LGSU01001248.1:5456-6394* | *Cyanophyta* | *Oscillatoriales* | *Solyc03g031860.2.1* |
| *Leptolyngbyaceae cyanobacterium* | *Lcy* | *360* | *2,00E-119* | *NJR49961* | *NJR49961* | *JAAUOZ010000055.1:27289-28224* | *Cyanophyta* | *Synechococcales* | *Solyc03g031860.2.1* |
| *Nostocales cyanobacterium* | *Ncy* | *348* | *3,00E-115* | *TAE59201* | *TAE59201* | *RDXV01000048.1:21825-22757* | *Cyanophyta* | *Nostocales* | *Solyc03g031860.2.1* |
| *Okeania sp.* | *Osp* | *348* | *5,00E-115* | *NES03100* | *NES03100* | *JAAHGT010000113.1:11469-12407* | *Cyanophyta* | *Oscillatoriales* | *Solyc03g031860.2.1* |
| *Oscillatoriales cyanobacterium* | *Ocy* | *349* | *1,00E-117* | *OIP71133.1* | *OIP71133.1* | | *Cyanophyta* | *Oscillatoriales* | *Solyc03g031860.2.1* |
| *Spirulinaceae cyanobacterium* | *Scy* | *348* | *4,00E-115* | *NJK99192* | *NJK99192* | *JAAUTY010000001.1:236214-237143* | *Cyanophyta* | *Spirulinales* | *Solyc03g031860.2.1* |
| *Synechococcus elongatus* | *Sel* | *365* | *1,00E-123* | *QFZ92142.1* | *QFZ92142.1* | | *Cyanophyta* | *Synechococcales* | *Solyc03g031860.2.1* |
| *Thermosynechococcus elongatus* | *Tel* | *352* | *7,00E-119* | *AXY68520.1* | *AXY68520.1* | | *Cyanophyta* | *Synechococcales* | *Solyc03g031860.2.1* |
| *Chlamydomonas reinhardtii* | *Cre* | *402.1* | *3.3E-137* | *Cre02.g095092.t1.1 (primary)* | *Cre02.g095092.t1.1* | *chromosome_2:3336147..3340314 reverse* | *Chlorophyta* | *Volvocales* | *Solyc03g031860.2.1* |

| Species | Acronymon | Score | Evalue | Transcript Name | CDS Acession Number | Location | Classification group | Order | Query |
|---|---|---|---|---|---|---|---|---|---|
| *Coccomyxa subellipsoidea* | *Csu* | *387.9* | *1,00E-132* | *52083 (primary)* | *52083* | *scaffold_1:571729..575130 forward* | *Chlorophyta* | *Incertae sedis* | *Solyc03g031860.2.1* |
| *Dunaliella salina* | *Das* | *404.8* | *6.3E-138* | *Dusal.0943s00001.1 (primary)* | *Dusal.0943s00001.1* | *scaffold943:9490..19698 reverse* | *Chlorophyta* | *Chlamydomonadales* | *Solyc03g031860.2.1* |
| *Dunaliella salina* | *Das* | *374.0* | *6.3E-126* | *Dusal.0031s00032.1 (primary)* | *Dusal.0031s00032.1* | *scaffold31:479535..492555 forward* | *Chlorophyta* | *Chlamydomonadales* | *Solyc03g031860.2.1* |
| *Micromonas pusilla* | *Mpu* | *385.6* | *1.5E-130* | *32317 (primary)* | *32317* | *scaffold_3:822114..824691 reverse* | *Chlorophyta* | *Mamiellales* | *Solyc03g031860.2.1* |
| *Micromonas sp* | *M* | *387.1* | *2.7E-132* | *85839 (primary)* | *85839* | *Chr_10:705277..706335 forward* | *Chlorophyta* | *Mamiellales* | *Solyc03g031860.2.1* |
| *Ostreococcus lucimarinus* | *Olu* | *349.7* | *7,00E-119* | *12559 (primary)* | *12559* | *Chr_5:565049..565870 reverse* | *Chlorophyta* | *Mamiellales* | *Solyc03g031860.2.1* |
| *Volvox carteri* | *Vca* | *402.5* | *2.2E-137* | *Vocar.0027s0175.1* | *Vocar.0027s0175.1* | *scaffold_27:1630403..1637580 reverse* | *Chlorophyta* | *Volvocales* | *Solyc03g031860.2.1* |
| *Marchantia polymorpha* | *Mpo* | *532.7* | *0.0* | *Mapoly0040s0104.1 (primary)* | *Mapoly0040s0104.1* | *scaffold_40:1067130..1071400 forward* | *Bryophyta* | *Marchantiales* | *Solyc03g031860.2.1* |
| *Physcomitrella patens* | *Ppa* | *531.6* | *0.0* | *Pp3c24_10180V3.1 (primary)* | *Pp3c24_10180V3.1* | *Chr24:6938725..6942585 forward* | *Bryophyta* | *Funariales* | *Solyc03g031860.2.1* |
| *Physcomitrella patens* | *Ppa* | *520* | *0.0* | *Pp3c8_21760V3.1 (primary)* | *Pp3c8_21760V3.1* | *Chr08:14919012..14922846 forward* | *Bryophyta* | *Funariales* | *Solyc03g031860.2.1* |
| *Physcomitrella patens* | *Ppa* | *518.9* | *0.0* | *Pp3c24_16380V3.1 (primary)* | *Pp3c24_16380V3.1* | *Chr24:10757319..10761131 forward* | *Bryophyta* | *Funariales* | *Solyc03g031860.2.1* |
| *Sphagnum fallax* | *Sfa* | *521.9* | *0.0* | *Sphfalx0049s0098.1 (primary)* | *Sphfalx0049s0098.1* | *super_49:1449572..1454449 reverse* | *Bryophyta* | *Sphagnales* | *Solyc03g031860.2.1* |
| *Aspergillus costaricaensis* | *Acs* | *82* | *7,00E-14* | *XP_025539973* | *XP_025539973* | | *Fungi* | *Eurotiales* | *Solyc03g031860.2.1* |
| *Aspergillus luchuensis* | *Alu* | *83,2* | *5,00E-15* | *GAT30047* | *GAT30047* | *map: 6-6 F, contig: AA1_SCon_033* | *Fungi* | *Eurotiales* | *Solyc03g031860.2.1* |
| *Aspergillus neoniger* | *Ane* | *80,9* | *2,00E-13* | *XP_025475515* | *XP_025475515* | | *Fungi* | *Eurotiales* | *Solyc03g031860.2.1* |
| *Aspergillus piperis* | *Api* | *83,2* | *5,00E-15* | *XP_025516982* | *XP_025516982* | | *Fungi* | *Eurotiales* | *Solyc03g031860.2.1* |
| *Colletotrichum higginsianum* | *Chi* | *75,1* | *8,00E-12* | *CCF46103* | *CCF46103* | *contig cont1.829* | *Fungi* | *Glomerellales* | *Solyc03g031860.2.1* |
| *Fusarium albosuccineum* | *Fal* | *77* | *1,00E-12* | *KAF4458563* | *KAF4458563* | | *Fungi* | *Hypocreales* | *Solyc03g031860.2.1* |

| Species | Acronymon | Score | Evalue | Transcript Name | CDS Acession Number | Location | Classification group | Order | Query |
|---|---|---|---|---|---|---|---|---|---|
| *Nannizzia gypsea* | *Ngy* | *80,9* | *2,00E-13* | *XP_003174566* | *XP_003174566* | | *Fungi* | *Onygenales* | *Solyc03g031860.2.1* |
| *Pyrenophora seminiperda* | *Pse* | *94,4* | *9,00E-18* | *RMZ72926* | *RMZ72926* | *scaffold00043* | *Fungi* | *Pleosporales* | *Solyc03g031860.2.1* |
| *Pyrenophora tritici-repentis* | *Pti* | *89* | *2,00E-16* | *KAA8614953* | *KAA8614953* | *V1_contig_00009* | *Fungi* | *Pleosporales* | *Solyc03g031860.2.1* |
| *Rosellinia necatrix* | *Rne* | *76,3* | *5,00E-12* | *GAP92111* | *GAP92111* | *scaffold: Contig75* | *Fungi* | *Xylariales* | *Solyc03g031860.2.1* |
| *Acyrthosiphon pisum* | *Aps* | *64,7* | *1,00E-08* | *XP_003241668* | *XP_003241668* | | *Arthropoda* | *Hemiptera* | *Solyc03g031860.2.1* |
| *Drosophila bipectinata* | *Dbi* | *93,6* | *6,00E-19* | *XP_017095802* | *XP_017095802* | | *Arthropoda* | *Diptera* | *Solyc03g031860.2.1* |
| *Drosophila elegans* | *Del* | *93,2* | *8,00E-19* | *XP_017118015* | *XP_017118015* | | *Arthropoda* | *Diptera* | *Solyc03g031860.2.1* |
| *Drosophila eugracilis* | *Deu* | *108* | *3,00E-24* | *XP_017070630* | *XP_017070630* | | *Arthropoda* | *Diptera* | *Solyc03g031860.2.1* |
| *Drosophila ficusphila* | *Dfi* | *109* | *7,00E-25* | *XP_017040643* | *XP_017040643* | | *Arthropoda* | *Diptera* | *Solyc03g031860.2.1* |
| *Drosophila kikkawai* | *Dki* | *93,2* | *7,00E-19* | *XP_017032848* | *XP_017032848* | | *Arthropoda* | *Diptera* | *Solyc03g031860.2.1* |
| *Tetranychus urticae* | *Tut* | *56* | *0.00035* | *tetur01g11260* | *DAA34969.1* | *SuperContig HE587301: 5,209,423-5,213,678 reverse strand* | *Arthropoda* | *Trombidiformes* | *Tetranychus urticae ASM23943v1* |
| *Klebsormidium nitens* | *Kni* | *448* | *3,00E-154* | *kfl00019_0320_v1.1* | *kfl00019_0320* | | *Charophyta* | *Klebsormidiales* | *Solyc03g031860.2.1* |
| *Selaginella moellendorffii* | *Smo* | *523.1* | *0.0* | *233658 (primary)* | *233658* | *scaffold_47:782646..784025 forward* | *Lycophytes* | *Selaginellales* | *Solyc03g031860.2.1* |
| *Picea abies* | *Pab* | *567,38* | *0.0* | *MA_86494g0010* | *MA_86494g0010* | *MA_86494:2318..6959* | *Gymnosperm* | *Pinales* | *Solyc03g031860.2.1* |
| *Picea abies* | *Pab* | *236,5* | *0.0* | *MA_407452g0010* | *MA_407452g0010* | *MA_407452:1629..2244* | *Gymnosperm* | *Pinales* | *Solyc03g031860.2.1* |
| *Pinus taeda* | *Pta* | *539,26* | *0.0* | *PITA_000050116* | *PITA_000050116* | *Scaffold 880084.2:2751..7208* | *Gymnosperm* | *Pinales* | *Solyc03g031860.2.1* |
| *Pinus taeda* | *Pta* | *480,71* | *9,83E-170* | *PITA_000002541* | *PITA_000002541* | *tscaffold 4861:26078..42463* | *Gymnosperm* | *Pinales* | *Solyc03g031860.2.1* |

| Species | Acronymon | Score | Evalue | Transcript Name | CDS Acession Number | Location | Classification group | Order | Query |
|---|---|---|---|---|---|---|---|---|---|
| *Amborella trichopoda* | *Atr* | *562* | *0.0* | *evm_27.model.AmTr_v1.0_scaffold00032.92 (primary)* | *evm_27.model.AmTr_v1.0_scaffold00032.92* | *AmTr_v1.0_scaffold00032:1823840..1832740 reverse* | *Early angiosperm* | *Amborellales* | *Solyc03g031860.2.1* |
| *Amborella trichopoda* | *Atr* | *537.3* | *0.0* | *evm_27.model.AmTr_v1.0_scaffold00044.14 (primary)* | *evm_27.model.AmTr_v1.0_scaffold00044.14* | *AmTr_v1.0_scaffold00044:320296..333892 reverse* | *Early angiosperm* | *Amborellales* | *Solyc03g031860.2.1* |
| *Spirodela polyrhiza* | *Spo* | *602.4* | *0.0* | *Spipo1G0051600 (primary)* | *Spipo1G0051600* | *pseudo1:3342204..3344393 forward* | *Monocots* | *Alismatales* | *Solyc03g031860.2.1* |
| *Spirodela polyrhiza* | *Spo* | *546.6* | *0.0* | *Spipo8G0020300 (primary)* | *Spipo8G0020300* | *pseudo8:2050614..2052383 reverse* | *Monocots* | *Alismatales* | *Solyc03g031860.2.1* |
| *Zostera marina* | *Zmr* | *569.7* | *0.0* | *Zosma16g00900.1* | *Zosma16g00900.1* | *scaffold_16:567834..570010 reverse* | *Monocots* | *Alismatales* | *Solyc03g031860.2.1* |
| *Aegilops tauschii* | *Ata* | *1276* | *3,00E-168* | *AET7Gv21342500.2* | *AET7Gv21342500* | *Chromosome 7D: 639,162,008-639,167,161 reverse strand.* | *Monocots (commelinids)* | *Poales* | *Solyc03g031860.2.1* |
| *Aegilops tauschii* | *Ata* | *1254* | *3,40E-165* | *AET5Gv20068500.3* | *AET5Gv20068500* | *Chromosome 5D: 26,757,085-26,760,088 reverse strand.* | *Monocots (commelinids)* | *Poales* | *Solyc03g031860.2.1* |
| *Aegilops tauschii* | *Ata* | *753* | *1,10E-95* | *AET5Gv20828700.1* | *AET5Gv20828700* | *Chromosome 5D: 450,481,493-450,484,002 forward strand* | *Monocots (commelinids)* | *Poales* | *Solyc03g031860.2.1* |
| *Ananas comosus* | *Aco* | *581.3* | *0.0* | *Aco013002.1 (primary)* | *Aco013002.1* | *LG25:1393795..1396471 reverse* | *Monocots (commelinids)* | *Poales* | *Solyc03g031860.2.1* |
| *Ananas comosus* | *Aco* | *575.5* | *0.0* | *Aco009217.1 (primary)* | *Aco009217.1* | *LG22:8546722..8558411 reverse* | *Monocots (commelinids)* | *Poales* | *Solyc03g031860.2.1* |
| *Ananas comosus* | *Aco* | *541.6* | *0.0* | *Aco005022.1 (primary)* | *Aco005022.1* | *LG07:1487658..1490439 forward* | *Monocots (commelinids)* | *Poales* | *Solyc03g031860.2.1* |
| *Brachypodium distachyon* | *Bdi* | *549.7* | *0.0* | *Bradi1g29590.2 (primary)* | *Bradi1g29590.2* | *Bd1:25177642..25181644 forward* | *Monocots (commelinids)* | *Poales* | *Solyc03g031860.2.1* |
| *Brachypodium distachyon* | *Bdi* | *510.0* | *3.8E-179* | *Bradi4g01100.1 (primary)* | *Bradi4g01100.1* | *Bd4:683690..687242 reverse* | *Monocots (commelinids)* | *Poales* | *Solyc03g031860.2.1* |
| *Brachypodium distachyon* | *Bdi* | *494.2* | *4.6E-173* | *Bradi4g37520.1 (primary)* | *Bradi4g37520.1* | *Bd4:42521097..42524510 reverse* | *Monocots (commelinids)* | *Poales* | *Solyc03g031860.2.1* |
| *Brachypodium stacei* | *Bst* | *554.3* | *0.0* | *Brast07G241400.1 (primary)* | *Brast07G241400.1* | *Chr07:20604273..20608120 reverse* | *Monocots (commelinids)* | *Poales* | *Solyc03g031860.2.1* |
| *Brachypodium stacei* | *Bst* | *513.1* | *1.3E-180* | *Brast10G009800.1 (primary)* | *Brast10G009800.1* | *Chr10:577285..580570 reverse* | *Monocots (commelinids)* | *Poales* | *Solyc03g031860.2.1* |

| Species | Acronymon | Score | Evalue | Transcript Name | CDS Acession Number | Location | Classification group | Order | Query |
|---|---|---|---|---|---|---|---|---|---|
| *Brachypodium stacei* | *Bst* | *488.4* | *9.2E-171* | *Brast05G209700.1 (primary)* | *Brast05G209700.1* | *Chr05:17783948..17786829 reverse* | *Monocots (commelinids)* | *Poales* | *Solyc03g031860.2.1* |
| *Eragrostis tef* | *Ete* | *391* | *7,70E-118* | *Et_s3092-0.38-1.mrna1* | *Et_s3092* | *SuperContig scaffold3092: 12,555-15,280 forward strand* | *Monocots (commelinids)* | *Poales* | *Solyc03g031860.2.1* |
| *Eragrostis tef* | *Ete* | *372* | *4,00E-103* | *Et_s1813-1.34-1.mrna1* | *Et_s1813* | *SuperContig scaffold1813: 173,520-176,190 forward strand* | *Monocots (commelinids)* | *Poales* | *Solyc03g031860.2.1* |
| *Eragrostis tef* | *Ete* | *371* | *8,10E-125* | *Et_s7091-0.40-1.mrna1* | *Et_s7091* | *SuperContig scaffold7091: 10,905-13,395 forward strand* | *Monocots (commelinids)* | *Poales* | *Solyc03g031860.2.1* |
| *Eragrostis tef* | *Ete* | *351* | *7,00E-128* | *Et_s517-0.16-1.mrna1* | *Et_s517* | *SuperContig scaffold517: 41,323-45,588 forward strand.* | *Monocots (commelinids)* | *Poales* | *Solyc03g031860.2.1* |
| *Eragrostis tef* | *Ete* | *349* | *1,20E-129* | *Et_s4159-0.20-1.mrna1* | *Et_s4159* | *SuperContig scaffold4159: 66,805-70,707 reverse strand* | *Monocots (commelinids)* | *Poales* | *Solyc03g031860.2.1* |
| *Hordeum vulgare* | *Hvu* | *1269* | *2,80E-167* | *HORVU7Hr1G120660.3* | *HORVU7Hr1G120660* | *Chromosome chr7H: 652,117,486-652,121,143 forward strand.* | *Monocots (commelinids)* | *Poales* | *Solyc03g031860.2.1* |
| *Hordeum vulgare* | *Hvu* | *1258* | *9,30E-166* | *HORVU5Hr1G005740.2* | *HORVU5Hr1G005740* | *Chromosome chr5H: 9,252,064-9,255,073 reverse strand* | *Monocots (commelinids)* | *Poales* | *Solyc03g031860.2.1* |
| *Hordeum vulgare* | *Hvu* | *750* | *2,90E-95* | *HORVU5Hr1G088130.4* | *HORVU5Hr1G088130* | *Chromosome chr5H: 580,830,866-580,832,556 forward strand* | *Monocots (commelinids)* | *Poales* | *Solyc03g031860.2.1* |
| *Leersia perrieri* | *Lpe* | *1309* | *1,70E-173* | *LPERR06G23640.1* | *LPERR06G23640* | *Chromosome 6: 20,892,913-20,895,557 reverse strand.* | *Monocots (commelinids)* | *Poales* | *Solyc03g031860.2.1* |
| *Leersia perrieri* | *Lpe* | *1259* | *1,50E-166* | *LPERR12G16110.1* | *LPERR12G16110* | *Chromosome 12: 16,999,952-17,002,773 reverse strand* | *Monocots (commelinids)* | *Poales* | *Solyc03g031860.2.1* |
| *Leersia perrieri* | *Lpe* | *794* | *4,90E-102* | *LPERR09G15010.1* | *LPERR09G15010* | *Chromosome 9: 14,625,116-14,627,798 reverse strand.* | *Monocots (commelinids)* | *Poales* | *Solyc03g031860.2.1* |
| *Leersia perrieri* | *Lpe* | *763* | *9,90E-98* | *LPERR09G15000.1* | *LPERR09G15000* | *Chromosome 9: 14,619,828-14,622,601 reverse strand* | *Monocots (commelinids)* | *Poales* | *Solyc03g031860.2.1* |
| *Musa acuminata* | *Mac* | *599* | *0.0* | *GSMUA_AchrUn_randomT09240_001 (primary)* | *GSMUA_AchrUn_randomT09240_001* | *chrUn_random:42933000..42935315 reverse* | *Monocots (commelinids)* | *Zingiberales* | *Solyc03g031860.2.1* |

| Species | Acronymon | Score | Evalue | Transcript Name | CDS Acession Number | Location | Classification group | Order | Query |
|---|---|---|---|---|---|---|---|---|---|
| *Musa acuminata* | *Mac* | *560.1* | *0.0* | *GSMUA_Achr9T10050_001 (primary)* | *GSMUA_Achr9T10050_001* | *chr9:6467097..6469206 forward* | *Monocots (commelinids)* | *Zingiberales* | *Solyc03g031860.2.1* |
| *Musa acuminata* | *Mac* | *537.3* | *0.0* | *GSMUA_Achr6T31560_001 (primary)* | *GSMUA_Achr6T31560_001* | *chr6:31294343..31298625 reverse* | *Monocots (commelinids)* | *Zingiberales* | *Solyc03g031860.2.1* |
| *Musa acuminata* | *Mac* | *527.3* | *0.0* | *GSMUA_Achr11T07450_001 (primary)* | *GSMUA_Achr11T07450_001* | *chr11:5827293..5829074 reverse* | *Monocots (commelinids)* | *Zingiberales* | *Solyc03g031860.2.1* |
| *Oropetium thomaeum* | *Oth* | *566.2* | *0.0* | *Oropetium_20150105_16450A (primary)* | *Oropetium_20150105_16450A* | *Oropetium_genomic_20141112_058:1394996..1399216 reverse* | *Monocots (commelinids)* | *Poales* | *Solyc03g031860.2.1* |
| *Oropetium thomaeum* | *Oth* | *501.1* | *9,00E-176* | *Oropetium_20150105_06439A (primary)* | *Oropetium_20150105_06439A* | *Oropetium_genomic_20141112_007:2298318..2301541 forward* | *Monocots (commelinids)* | *Poales* | *Solyc03g031860.2.1* |
| *Oropetium thomaeum* | *Oth* | *435.3* | *3.2E-2* | *Oropetium_20150105_26131A (primary)* | *Oropetium_20150105_26131A* | *Oropetium_genomic_20141112_128:102591..105980 reverse* | *Monocots (commelinids)* | *Poales* | *Solyc03g031860.2.1* |
| *Oryza barthii* | *Oba* | *1304* | *7,90E-173* | *OBART06G28180.1* | *OBART06G28180* | *Chromosome 6: 25,547,527-25,550,534 reverse strand.* | *Monocots (commelinids)* | *Poales* | *Solyc03g031860.2.1* |
| *Oryza barthii* | *Oba* | *1250* | *2,50E-165* | *OBART12G19460.1* | *OBART12G19460* | *Chromosome 12: 19,871,917-19,874,707 reverse strand* | *Monocots (commelinids)* | *Poales* | *Solyc03g031860.2.1* |
| *Oryza barthii* | *Oba* | *785* | *8,40E-101* | *OBART09G19150.1* | *OBART09G19150* | *Chromosome 9: 18,232,450-18,235,912 reverse strand.* | *Monocots (commelinids)* | *Poales* | *Solyc03g031860.2.1* |
| *Oryza brachyantha* | *Obr* | *1291* | *3,30E-171* | *OB06G35940.1* | *OB06G35940* | *Chromosome 6: 21,655,842-21,658,741 reverse strand.* | *Monocots (commelinids)* | *Poales* | *Solyc03g031860.2.1* |
| *Oryza brachyantha* | *Obr* | *1131* | *5,40E-149* | *OB12G26220.1* | *OB12G26220* | *Chromosome 12: 14,756,519-14,759,775 forward strand.* | *Monocots (commelinids)* | *Poales* | *Solyc03g031860.2.1* |
| *Oryza brachyantha* | *Obr* | *1076* | *2,30E-141* | *OB09G25760.1* | *OB09G25760* | *Chromosome 9: 13,346,492-13,348,554 reverse strand.* | *Monocots (commelinids)* | *Poales* | *Solyc03g031860.2.1* |
| *Oryza glaberrima* | *Ogl* | *1304* | *5,60E-173* | *ORGLA06G0287600.1* | *ORGLA06G0287600* | *Scaffold Oglab06_unplaced183: 46,183-49,189 reverse strand.* | *Monocots (commelinids)* | *Poales* | *Solyc03g031860.2.1* |

| Species | Acronymon | Score | Evalue | Transcript Name | CDS Acession Number | Location | Classification group | Order | Query |
|---|---|---|---|---|---|---|---|---|---|
| *Oryza glaberrima* | *Ogl* | *1256* | *2,60E-166* | *ORGLA12G0167800.1* | *ORGLA12G0167800* | *Chromosome 12: 18,361,617-18,364,404 reverse strand.* | *Monocots (commelinids)* | *Poales* | *Solyc03g031860.2.1* |
| *Oryza glaberrima* | *Ogl* | *1200* | *1,50E-158* | *ORGLA09G0153600.1* | *ORGLA09G0153600* | *Chromosome 9: 16,481,944-16,484,415 reverse strand* | *Monocots (commelinids)* | *Poales* | *Solyc03g031860.2.1* |
| *Oryza glumipatula* | *Ogu* | *1304* | *9,10E-173* | *OGLUM06G29570.1* | *OGLUM06G29570* | *Chromosome 6: 31,354,021-31,357,030 reverse strand* | *Monocots (commelinids)* | *Poales* | *Solyc03g031860.2.1* |
| *Oryza glumipatula* | *Ogu* | *1250* | *2,80E-165* | *OGLUM12G21130.1* | *OGLUM12G21130* | *Chromosome 12: 25,999,396-26,003,333 forward strand.* | *Monocots (commelinids)* | *Poales* | *Solyc03g031860.2.1* |
| *Oryza glumipatula* | *Ogu* | *694* | *4,10E-88* | *OGLUM09G19690.1* | *OGLUM09G19690* | *Chromosome 9: 22,265,018-22,267,694 reverse strand.* | *Monocots (commelinids)* | *Poales* | *Solyc03g031860.2.1* |
| *Oryza longistaminata* | *Olo* | *1311* | *5,80E-174* | *KN538785.1_FGT003* | *KN538785* | *Scaffold KN538785.1: 152,026-154,808 reverse strand* | *Monocots (commelinids)* | *Poales* | *Solyc03g031860.2.1* |
| *Oryza longistaminata* | *Olo* | *1088* | *5,10E-143* | *KN538757.1_FGT004* | *KN538757* | *Scaffold KN538757.1: 172,790-184,307 forward strand.* | *Monocots (commelinids)* | *Poales* | *Solyc03g031860.2.1* |
| *Oryza longistaminata* | *Olo* | *764* | *4,70E-98* | *KN540821.1_FGT003* | *KN540821* | *Scaffold KN540821.1: 12,579-18,354 forward strand* | *Monocots (commelinids)* | *Poales* | *Solyc03g031860.2.1* |
| *Oryza meridionalis* | *Ome* | *1304* | *8,70E-173* | *OMERI06G28090.5* | *OMERI06G28090* | *Chromosome 6: 31,628,915-31,633,028 reverse strand.* | *Monocots (commelinids)* | *Poales* | *Solyc03g031860.2.1* |
| *Oryza meridionalis* | *Ome* | *1195* | *1,20E-157* | *OMERI09G14390.1* | *OMERI09G14390* | *Chromosome 9: 17,923,693-17,929,959 reverse strand.* | *Monocots (commelinids)* | *Poales* | *Solyc03g031860.2.1* |
| *Oryza meridionalis* | *Ome* | *1180* | *1,40E-155* | *OMERI12G14330.1* | *OMERI12G14330* | *Chromosome 12: 19,269,363-19,282,654 reverse strand.* | *Monocots (commelinids)* | *Poales* | *Solyc03g031860.2.1* |
| *Oryza punctata* | *Opu* | *1306* | *4,50E-173* | *OPUNC06G25700.2* | *OPUNC06G25700* | *Chromosome 6: 34,382,050-34,384,709 reverse strand.* | *Monocots (commelinids)* | *Poales* | *Solyc03g031860.2.1* |
| *Oryza punctata* | *Opu* | *1206* | *1,10E-166* | *OPUNC12G17610.1* | *OPUNC12G17610* | *Chromosome 12: 27,114,787-27,119,055 forward strand.* | *Monocots (commelinids)* | *Poales* | *Solyc03g031860.2.1* |
| *Oryza punctata* | *Opu* | *1201* | *1,70E-158* | *OPUNC09G17510.1* | *OPUNC09G17510* | *Chromosome 9: 25,407,635-25,409,731 reverse strand.* | *Monocots (commelinids)* | *Poales* | *Solyc03g031860.2.1* |
| *Oryza rufipogon* | *Oru* | *1304* | *9,30E-173* | *ORUFI06G30110.1* | *ORUFI06G30110* | *Chromosome 6: 27,682,342-27,686,178 reverse strand.* | *Monocots (commelinids)* | *Poales* | *Solyc03g031860.2.1* |

| Species | Acronymon | Score | Evalue | Transcript Name | CDS Acession Number | Location | Classification group | Order | Query |
|---|---|---|---|---|---|---|---|---|---|
| *Oryza rufipogon* | *Oru* | *1205* | *2,90E-165* | *ORUFI12G21740.1* | *ORUFI12G21740* | *Chromosome 12: 22,831,897-22,834,687 reverse strand.* | *Monocots (commelinids)* | *Poales* | *Solyc03g031860.2.1* |
| *Oryza rufipogon* | *Oru* | *793* | *7,60E-102* | *ORUFI09G20730.1* | *ORUFI09G20730* | *Chromosome 9: 19,611,150-19,617,449 reverse strand* | *Monocots (commelinids)* | *Poales* | *Solyc03g031860.2.1* |
| *Oryza sativa* | *Osa* | *563.1* | *0.0* | *LOC_Os06g51290.1 (primary)* | *LOC_Os06g51290.1* | *Chr6:31051971..31055926 reverse* | *Monocots (commelinids)* | *Poales* | *Solyc03g031860.2.1* |
| *Oryza sativa* | *Osa* | *514.6* | *0.0* | *LOC_Os12g43130.1 (primary)* | *LOC_Os12g43130.1* | *Chr12:26780487..26783598 reverse* | *Monocots (commelinids)* | *Poales* | *Solyc03g031860.2.1* |
| *Oryza sativa* | *Osa* | *491.5* | *3.3E-171* | *LOC_Os09g38320.1 (primary)* | *LOC_Os09g38320.1* | *Chr9:22041967..22045393 reverse* | *Monocots (commelinids)* | *Poales* | *Solyc03g031860.2.1* |
| *Panicum hallii* | *Pha* | *572.0* | *0.0* | *Pahal.D00247.1 (primary)* | *Pahal.D00247.1* | *Chr04:226949..230520 forward* | *Monocots (commelinids)* | *Poales* | *Solyc03g031860.2.1* |
| *Panicum hallii* | *Pha* | *517.3* | *0.0* | *Pahal.C04830.1 (primary)* | *Pahal.C04830.1* | *Chr03:63469691..63473041 forward* | *Monocots (commelinids)* | *Poales* | *Solyc03g031860.2.1* |
| *Panicum hallii* | *Pha* | *507.7* | *1,00E-177* | *Pahal.B03778.1 (primary)* | *Pahal.B03778.1* | *Chr02:52670089..52674217 reverse* | *Monocots (commelinids)* | *Poales* | *Solyc03g031860.2.1* |
| *Panicum virgatum* | *Pvi* | *575.1* | *0.0* | *Pavir.J02631.1 (primary)* | *Pavir.J02631.1* | *contig03129:15958..19364 reverse* | *Monocots (commelinids)* | *Poales* | *Solyc03g031860.2.1* |
| *Panicum virgatum* | *Pvi* | *569.3* | *0.0* | *Pavir.Db00050.1 (primary)* | *Pavir.Db00050.1* | *Chr04b:736370..740005 reverse* | *Monocots (commelinids)* | *Poales* | *Solyc03g031860.2.1* |
| *Panicum virgatum* | *Pvi* | *518.5* | *0.0* | *Pavir.Db01083.1 (primary)* | *Pavir.Db01083.1* | *Chr04b:19360788..19363716 forward* | *Monocots (commelinids)* | *Poales* | *Solyc03g031860.2.1* |
| *Panicum virgatum* | *Pvi* | *501.5* | *2.4E-176* | *Pavir.Ba01490.1 (primary)* | *Pavir.Ba01490.1* | *Chr02a:19032833..19035996 reverse* | *Monocots (commelinids)* | *Poales* | *Solyc03g031860.2.1* |
| *Panicum virgatum* | *Pvi* | *490.3* | *1.1E-172* | *Pavir.Bb02573.1 (primary)* | *Pavir.Bb02573.1* | *Chr02b:59338774..59341023 reverse* | *Monocots (commelinids)* | *Poales* | *Solyc03g031860.2.1* |
| *Panicum virgatum* | *Pvi* | *455.7* | *1.4E-159* | *Pavir.Cb00105.1 (primary)* | *Pavir.Cb00105.1* | *Chr03b:1647112..1649439 forward* | *Monocots (commelinids)* | *Poales* | *Solyc03g031860.2.1* |
| *Saccharum spontaneum* | *Ssp* | *384* | *2,50E-125* | *Sspon.08G0000480-1A-mRNA-1* | *Sspon_08G0000480* | *Chromosome 8A: 1,745,103-1,748,728 forward strand* | *Monocots (commelinids)* | *Poales* | *Solyc03g031860.2.1* |
| *Setaria italica* | *Sit* | *570.5* | *0.0* | *Seita.4G288600.1 (primary)* | *Seita.4G288600.1* | *scaffold_4:40043909..40046902 reverse* | *Monocots (commelinids)* | *Poales* | *Solyc03g031860.2.1* |
| *Setaria italica* | *Sit* | *519.2* | *0.0* | *Seita.3G397800.1 (primary)* | *Seita.3G397800.1* | *scaffold_3:49831722..49834779 forward* | *Monocots (commelinids)* | *Poales* | *Solyc03g031860.2.1* |
| *Setaria italica* | *Sit* | *503.8* | *3.4E-176* | *Seita.2G303000.1 (primary)* | *Seita.2G303000.1* | *scaffold_2:39561335..39564733 reverse* | *Monocots (commelinids)* | *Poales* | *Solyc03g031860.2.1* |

| Species | Acronymon | Score | Evalue | Transcript Name | CDS Acession Number | Location | Classification group | Order | Query |
|---|---|---|---|---|---|---|---|---|---|
| *Setaria viridis* | *Svi* | *568.2* | *0.0* | *Sevir.4G300900.1 (primary)* | *Sevir.4G300900.1* | *Chr_04:39324444..39327897 reverse* | *Monocots (commelinids)* | *Poales* | *Solyc03g031860.2.1* |
| *Setaria viridis* | *Svi* | *518.5* | *0.0* | *Sevir.3G415800.1 (primary)* | *Sevir.3G415800.1* | *Chr_03:48966579..48969560 forward* | *Monocots (commelinids)* | *Poales* | *Solyc03g031860.2.1* |
| *Setaria viridis* | *Svi* | *504.6* | *2.1E-176* | *Sevir.2G314000.1 (primary)* | *Sevir.2G314000.1* | *Chr_02:38422397..38427143 reverse* | *Monocots (commelinids)* | *Poales* | *Solyc03g031860.2.1* |
| *Sorghum bicolor* | *Sbi* | *526.2* | *0.0* | *Sobic.008G180800.1 (primary)* | *Sobic.008G180800.1* | *Chr08:61419851..61422820 forward* | *Monocots (commelinids)* | *Poales* | *Solyc03g031860.2.1* |
| *Sorghum bicolor* | *Sbi* | *508.8* | *4.9E-178* | *Sobic.002G292600.1 (primary)* | *Sobic.002G292600.1* | *Chr02:67031565..67034288 reverse* | *Monocots (commelinids)* | *Poales* | *Solyc03g031860.2.1* |
| *Triticum aestivum* | *Tae* | *1276* | *2,10E-168* | *TraesCS7D02G553300.1* | *TraesCS7D02G553300* | *Chromosome 7D: 636,765,931-636,771,068 forward strand* | *Monocots (commelinids)* | *Poales* | *Solyc03g031860.2.1* |
| *Triticum aestivum* | *Tae* | *1273* | *5,60E-168* | *TraesCS7A02G557300.2* | *TraesCS7A02G557300* | *Chromosome 7A: 729,397,165-729,401,834 reverse strand.* | *Monocots (commelinids)* | *Poales* | *Solyc03g031860.2.1* |
| *Triticum aestivum* | *Tae* | *1270* | *1,50E-167* | *TraesCS7B02G482000.1* | *TraesCS7B02G482000* | *Chromosome 7B: 739,442,097-739,446,001 reverse strand* | *Monocots (commelinids)* | *Poales* | *Solyc03g031860.2.1* |
| *Triticum aestivum* | *Tae* | *1254* | *2,40E-165* | *TraesCS5D02G026000.1* | *TraesCS5D02G026000* | *Chromosome 5D: 23,296,528-23,299,514 reverse strand.* | *Monocots (commelinids)* | *Poales* | *Solyc03g031860.2.1* |
| *Triticum aestivum* | *Tae* | *1254* | *2,40E-165* | *TraesCS5B02G017900.1* | *TraesCS5B02G017900* | *Chromosome 5B: 17,544,779-17,547,737 reverse strand.* | *Monocots (commelinids)* | *Poales* | *Solyc03g031860.2.1* |
| *Triticum aestivum* | *Tae* | *1251* | *6,30E-165* | *TraesCS5A02G020900.1* | *TraesCS5A02G020900* | *Chromosome 5A: 17,221,404-17,224,338 reverse strand.* | *Monocots (commelinids)* | *Poales* | *Solyc03g031860.2.1* |
| *Triticum aestivum* | *Tae* | *763* | *3,30E-97* | *TraesCS5A02G356300.2* | *TraesCS5A02G356300* | *Chromosome 5A: 558,682,954-558,688,761 forward strand* | *Monocots (commelinids)* | *Poales* | *Solyc03g031860.2.1* |
| *Triticum aestivum* | *Tae* | *758* | *1,60E-96* | *TraesCS5B02G358800.1* | *TraesCS5B02G358800* | *Chromosome 5B: 538,717,321-538,719,110 forward strand.* | *Monocots (commelinids)* | *Poales* | *Solyc03g031860.2.1* |
| *Triticum aestivum* | *Tae* | *753* | *8,10E-96* | *TraesCS5D02G365100.1* | *TraesCS5D02G365100* | *Chromosome 5D: 442,496,502-442,498,524 forward strand* | *Monocots (commelinids)* | *Poales* | *Solyc03g031860.2.1* |

| Species | Acronymon | Score | Evalue | Transcript Name | CDS Acession Number | Location | Classification group | Order | Query |
|---|---|---|---|---|---|---|---|---|---|
| *Triticum dicoccoides* | *Tdi* | *1273* | *9,10E-168* | *TRIDC7AG077320.6* | *TRIDC7AG077320* | *Chromosome 7A: 721,057,396-721,061,077 reverse strand* | *Monocots (commelinids)* | *Poales* | *Solyc03g031860.2.1* |
| *Triticum dicoccoides* | *Tdi* | *1259* | *7,90E-166* | *TRIDC7BG073780.3* | *TRIDC7BG073780* | *Chromosome 7B: 739,010,245-739,013,465 forward strand.* | *Monocots (commelinids)* | *Poales* | *Solyc03g031860.2.1* |
| *Triticum dicoccoides* | *Tdi* | *1251* | *1,00E-164* | *TRIDC5AG002900.4* | *TRIDC5AG002900* | *Chromosome 5A: 16,297,965-16,300,537 reverse strand.* | *Monocots (commelinids)* | *Poales* | *Solyc03g031860.2.1* |
| *Triticum dicoccoides* | *Tdi* | *758* | *2,70E-96* | *TRIDC5BG056420.1* | *TRIDC5BG056420* | *Chromosome 5B: 544,909,913-544,911,433 forward strand.* | *Monocots (commelinids)* | *Poales* | *Solyc03g031860.2.1* |
| *Triticum dicoccoides* | *Tdi* | *677* | *4,70E-85* | *TRIDC5AG052060.3* | *TRIDC5AG052060* | *Chromosome 5A: 554,272,803-554,273,524 forward strand* | *Monocots (commelinids)* | *Poales* | *Solyc03g031860.2.1* |
| *Triticum turgidum* | *Ttu* | *1281* | *6,60E-169* | *TRITD6Bv1G228570.1* | *TRITD6Bv1G228570* | *Chromosome 6B: 696,979,833-696,983,031 forward strand* | *Monocots (commelinids)* | *Poales* | *Solyc03g031860.2.1* |
| *Triticum turgidum* | *Ttu* | *1147* | *2,60E-150* | *TRITD5Bv1G006790.1* | *TRITD5Bv1G006790* | *Chromosome 5B: 17,326,775-17,328,203 forward strand* | *Monocots (commelinids)* | *Poales* | *Solyc03g031860.2.1* |
| *Triticum turgidum* | *Ttu* | *1142* | *1,30E-149* | *TRITD5Av1G008310.1* | *TRITD5Av1G008310* | *Chromosome 5A: 17,616,395-17,617,884 reverse strand* | *Monocots (commelinids)* | *Poales* | *Solyc03g031860.2.1* |
| *Triticum turgidum* | *Ttu* | *1126* | *2,10E-147* | *TRITD5Bv1G006680.1* | *TRITD5Bv1G006680* | *Chromosome 5B: 17,026,598-17,106,302 reverse strand.* | *Monocots (commelinids)* | *Poales* | *Solyc03g031860.2.1* |
| *Triticum urartu* | *Tur* | *1079* | *9,50E-142* | *TRIUR3_33626-T1* | *TRIUR3_33626* | *SuperContig scaffold269090: 29,367-31,794 reverse strand* | *Monocots (commelinids)* | *Poales* | *Solyc03g031860.2.1* |
| *Triticum urartu* | *Tur* | *731* | *1,90E-93* | *TRIUR3_13950-T1* | *TRIUR3_13950* | *SuperContig scaffold52601: 21,221-22,002 reverse strand.* | *Monocots (commelinids)* | *Poales* | *Solyc03g031860.2.1* |
| *Zea mays* | *Zma* | *563.5* | *0.0* | *Zm00008a024386_T01* | *Zm00008a024386_T01* | *chr06:81555811..81559359 forward* | *Monocots (commelinids)* | *Poales* | *Solyc03g031860.2.1* |
| *Zea mays* | *Zma* | *515.4* | *0.0* | *Zm00008a032909_T01* | *Zm00008a032909_T01* | *chr08:168204484..168208257 reverse* | *Monocots (commelinids)* | *Poales* | *Solyc03g031860.2.1* |

| Species | Acronymon | Score | Evalue | Transcript Name | CDS Acession Number | Location | Classification group | Order | Query |
|---|---|---|---|---|---|---|---|---|---|
| *Zea mays* | *Zma* | *335.9* | *6.4E-112* | *Zm00008a028762_T01* | *Zm00008a028762_T01* | *chr07:146291552..146293434 forward* | *Monocots (commelinids)* | *Poales* | *Solyc03g031860.2.1* |
| *Zea mays (Ensembl)* | *Zma* | *518.5* | *0.0* | *GRMZM2G149317_T01* | *GRMZM2G149317_T01* | *8:167823550..167826600 reverse* | *Monocots (commelinids)* | *Poales* | *Solyc03g031860.2.1* |
| *Zea mays (Ensembl)* | *Zma* | *345.9* | *1.3E-117* | *GRMZM2G300348_T01* | *GRMZM2G300348_T01* | *6:82182364..82184345 forward* | *Monocots (commelinids)* | *Poales* | *Solyc03g031860.2.1* |
| *Actinidia chinensis* | *Ach* | *1555* | *0.0* | *PSS00264* | *PSS00264* | *Chromosome LG21: 12,933,111-12,937,550 reverse strand* | *Eudicot (asterids)* | *Ericales* | *Solyc03g031860.2.1* |
| *Actinidia chinensis* | *Ach* | *1553* | *0.0* | *PSS30087* | *PSS30087* | *Chromosome LG5: 3,897,808-3,901,724 forward strand* | *Eudicot (asterids)* | *Ericales* | *Solyc03g031860.2.1* |
| *Actinidia chinensis* | *Ach* | *894* | *5,30E-116* | *PSR86897* | *PSR86897* | *Chromosome LG28: 15,339,888-15,343,821 reverse strand* | *Eudicot (asterids)* | *Ericales* | *Solyc03g031860.2.1* |
| *Aquilegia coerulea* | *Ace* | *570.9* | *0.0* | *Aqcoe3G009300.1 (primary)* | *Aqcoe3G009300.1* | *Chr_03:602263..605497 reverse* | *Eudicots* | *Ranunculales* | *Solyc03g031860.2.1* |
| *Aquilegia coerulea* | *Ace* | *527.7* | *0.0* | *Aqcoe1G178400.1 (primary)* | *Aqcoe1G178400.1* | *Chr_01:10395407..10398959 forward* | *Eudicots* | *Ranunculales* | *Solyc03g031860.2.1* |
| *Aquilegia coerulea* | *Ace* | *442.6* | *7.1E-153* | *Aqcoe1G050500.1 (primary)* | *Aqcoe1G050500.1* | *Chr_01:2555174..2556931 reverse* | *Eudicots* | *Ranunculales* | *Solyc03g031860.2.1* |
| *Coffea canephora* | *Cca* | *1566* | *0.0* | *CDP21283* | *CDP21283* | *SuperContig scaffold_3092: 6,986-12,616 reverse strand* | *Eudicots (asterids)* | *Gentianales* | *Solyc03g031860.2.1* |
| *Coffea canephora* | *Cca* | *1195* | *6,50E-158* | *CDP08373* | *CDP08373* | *Chromosome 8: 26,570,827-26,572,985 reverse strand* | *Eudicots (asterids)* | *Gentianales* | *Solyc03g031860.2.1* |
| *Coffea canephora* | *Cca* | *1101* | *7,20E-145* | *CDP17352* | *CDP17352* | *Chromosome 1: 4,751,718-4,753,782 reverse strand* | *Eudicots (asterids)* | *Gentianales* | *Solyc03g031860.2.1* |
| *Cynara cardunculus* | *Ccr* | *1581* | *0.0* | *KVI08675* | *KVI08675* | *SuperContig scaffold_374: 148,566-152,979 reverse strand* | *Eudicots (asterids)* | *Asterales* | *Solyc03g031860.2.1* |
| *Cynara cardunculus* | *Ccr* | *1126* | *2,60E-148* | *KVH90844* | *KVH90844* | *SuperContig scaffold_62: 345,600-347,490 reverse strand* | *Eudicots (asterids)* | *Asterales* | *Solyc03g031860.2.1* |
| *Cynara cardunculus* | *Ccr* | *780* | *2,70E-100* | *KVI03037* | *KVI03037* | *SuperContig scaffold_2482: 24,347-27,848 reverse strand* | *Eudicots (asterids)* | *Asterales* | *Solyc03g031860.2.1* |
| *Daucus carota* | *Dca* | *641.3* | *0.0* | *DCAR_010057 (primary)* | *DCAR_010057* | *DCARv2_Chr3:14246823..14250604 reverse* | *Eudicots (asterids)* | *Apiales* | *Solyc03g031860.2.1* |

| Species | Acronymon | Score | Evalue | Transcript Name | CDS Acession Number | Location | Classification group | Order | Query |
|---|---|---|---|---|---|---|---|---|---|
| *Daucus carota* | *Dca* | *530.0* | *0.0* | *DCAR_023043 (primary)* | *DCAR_023043* | *DCARv2_Chr6:2377864..2379676 forward* | *Eudicots (asterids)* | *Apiales* | *Solyc03g031860.2.1* |
| *Daucus carota* | *Dca* | *447.2* | *6,00E-155* | *DCAR_024333 (primary)* | *DCAR_024333* | *DCARv2_Chr7:12326481..12328432 forward* | *Eudicots (asterids)* | *Apiales* | *Solyc03g031860.2.1* |
| *Helianthus annuus* | *Han* | *392* | *6,40E-156* | *PSY1-1 OTG04734* | *OTG04734* | *Chromosome 12: 39,201,022-39,205,788 reverse strand* | *Eudicots (asterids)* | *Asterales* | *Solyc03g031860.2.1* |
| *Helianthus annuus* | *Han* | *378* | *2,60E-137* | *PSY-1 OTG06350* | *OTG06350* | *Chromosome 12: 159,042,358-159,047,933 reverse strand* | *Eudicots (asterids)* | *Asterales* | *Solyc03g031860.2.1* |
| *Helianthus annuus* | *Han* | *353* | *1,80E-95* | *OTG35442* | *OTG35442* | *Chromosome 2: 171,868,581-171,871,894 reverse strand* | *Eudicots (asterids)* | *Asterales* | *Solyc03g031860.2.1* |
| *Ipomoea triloba* | *Itr* | *393* | *3,50E-162* | *itb03g05110.t1* | *itb03g05110* | *Chromosome 3: 3,439,071-3,443,392 forward strand* | *Eudicots (asterids)* | *Solanales* | *Solyc03g031860.2.1* |
| *Ipomoea triloba* | *Itr* | *391* | *1,70E-136* | *itb12g01910.t1* | *itb12g01910* | *Chromosome 12: 1,291,564-1,295,742 forward strand* | *Eudicots (asterids)* | *Solanales* | *Solyc03g031860.2.1* |
| *Ipomoea triloba* | *Itr* | *327* | *3,80E-110* | *itb14g11400.t1* | *itb14g11400* | *Chromosome 14: 13,043,653-13,045,702 forward strand* | *Eudicots (asterids)* | *Solanales* | *Solyc03g031860.2.1* |
| *Mimulus guttatus* | *Mgu* | *646.7* | *0.0* | *Migut.K00976.1 (primary)* | *Migut.K00976.1* | *scaffold_11:14010950..14014009 forward* | *Eudicots (asterids)* | *Lamiales* | *Solyc03g031860.2.1* |
| *Mimulus guttatus* | *Mgu* | *458.8* | *2.5E-159* | *Migut.O00865.1 (primary)* | *Migut.O00865.1* | *scaffold_2956:1797..3477 forward* | *Eudicots (asterids)* | *Lamiales* | *Solyc03g031860.2.1* |
| *Mimulus guttatus* | *Mgu* | *458.0* | *5.4E-159* | *Migut.D02541.1 (primary)* | *Migut.D02541.1* | *scaffold_4:20917941..20919586 reverse* | *Eudicots (asterids)* | *Lamiales* | *Solyc03g031860.2.1* |
| *Nicotiana attenuata* | *Nat* | *1627* | *0.0* | *OIT35874* | *OIT35874* | *SuperContig scaffold00503: 205,508-208,871 reverse strand* | *Eudicots (asterids)* | *Solanales* | *Solyc03g031860.2.1* |
| *Nicotiana attenuata* | *Nat* | *1591* | *0.0* | *OIT34597* | *OIT34597* | *SuperContig scaffold00685: 31,867-35,812 reverse strand.* | *Eudicots (asterids)* | *Solanales* | *Solyc03g031860.2.1* |
| *Nicotiana attenuata* | *Nat* | *1132* | *4,30E-149* | *OIT32024* | *OIT32024* | *SuperContig scaffold01147: 16,867-20,125 reverse strand.* | *Eudicots (asterids)* | *Solanales* | *Solyc03g031860.2.1* |
| *Solanum lycopersicum* | *Sly* | *852.4* | *0.0* | *Solyc03g031860.2.1 (primary)* | *Solyc03g031860.2.1* | *SL2.50ch03:4326134..4330127 forward* | *Eudicots (asterids)* | *Solanales* | *Solyc03g031860.2.1* |

| Species | Acronymon | Score | Evalue | Transcript Name | CDS Acession Number | Location | Classification group | Order | Query |
|---|---|---|---|---|---|---|---|---|---|
| *Solanum lycopersicum* | *Sly* | *682.6* | *0.0* | *Solyc02g081330.2.1 (primary)* | *Solyc02g081330.2.1* | *SL2.50ch02:45335358..45339157 forward* | *Eudicots (asterids)* | *Solanales* | *Solyc03g031860.2.1* |
| *Solanum lycopersicum* | *Sly* | *449.5* | *8.7E-156* | *Solyc01g005940.2.1 (primary)* | *Solyc01g005940.2.1* | *SL2.50ch01:613955..617183 forward* | *Eudicots (asterids)* | *Solanales* | *Solyc03g031860.2.1* |
| *Solanum tuberosum* | *Stu* | *826.2* | *0.0* | *PGSC0003DMT400061846 (primary)* | *PGSC0003DMT400061846* | *ST4.03ch03:2742065..2744858 forward* | *Eudicots (asterids)* | *Solanales* | *Solyc03g031860.2.1* |
| *Solanum tuberosum* | *Stu* | *778.5* | *0.0* | *PGSC0003DMT400061845* | *GSC0003DMT400061845* | *ST4.03ch03:2742065..2744858 forward* | *Eudicots (asterids)* | *Solanales* | *Solyc03g031860.2.1* |
| *Linum usitatissimum* | *Lus* | *525.8* | *0.0* | *Lus10001416 (primary)* | *Lus10001416* | *scaffold85:30097..32075 reverse* | *Eudicots (rosid)* | *Malpighiales* | *Solyc03g031860.2.1* |
| *Linum usitatissimum* | *Lus* | *509.2* | *7.6E-179* | *Lus10001050 (primary)* | *Lus10001050* | *scaffold964:24886..26882 reverse* | *Eudicots (rosid)* | *Malpighiales* | *Solyc03g031860.2.1* |
| *Linum usitatissimum* | *Lus* | *476.9* | *1.8E-166* | *Lus10020729 (primary)* | *Lus10020729* | *scaffold303:421222..428724 reverse* | *Eudicots (rosid)* | *Malpighiales* | *Solyc03g031860.2.1* |
| *Linum usitatissimum* | *Lus* | *478.8* | *1.2E-165* | *Lus10029809 (primary)* | *Lus10029809* | *scaffold416:121008..122994 reverse* | *Eudicots (rosid)* | *Malpighiales* | *Solyc03g031860.2.1* |
| *Linum usitatissimum* | *Lus* | *445.7* | *4.5E-154* | *Lus10013753 (primary)* | *Lus10013753* | *scaffold1168:335239..337750 forward* | *Eudicots (rosid)* | *Malpighiales* | *Solyc03g031860.2.1* |
| *Linum usitatissimum* | *Lus* | *438.7* | *3,00E-151* | *Lus10039186 (primary)* | *Lus10039186* | *scaffold34:2009013..2011210 reverse* | *Eudicots (rosid)* | *Malpighiales* | *Solyc03g031860.2.1* |
| *Lupinus angustifolius* | *Lan* | *471* | *4,10E-147* | *OIW22018* | *OIW22018* | *SuperContig Scaffold_702: 7,016-9,463 reverse strand.* | *Eudicots (rosid)* | *Fabales* | *Solyc03g031860.2.1* |
| *Lupinus angustifolius* | *Lan* | *385* | *1,00E-121* | *OIW02629* | *OIW02629* | *Chromosome LG11: 2,451,597-2,454,165 forward strand* | *Eudicots (rosid)* | *Fabales* | *Solyc03g031860.2.1* |
| *Lupinus angustifolius* | *Lan* | *377* | *1,00E-120* | *OIW16120* | *OIW16120* | *Chromosome LG02: 21,937,526-21,941,060 reverse strand* | *Eudicots (rosid)* | *Fabales* | *Solyc03g031860.2.1* |
| *Malus domestica* | *Mdo* | *536.6* | *0.0* | *MDP0000237124 (primary)* | *MDP0000237124* | *MDC004033.136:2450..5320 reverse* | *Eudicots (rosid)* | *Rosales* | *Solyc03g031860.2.1* |
| *Malus domestica* | *Mdo* | *484.6* | *4.7E-169* | *MDP0000177623 (primary)* | *MDP0000177623* | *MDC008336.467:13749..16522 reverse* | *Eudicots (rosid)* | *Rosales* | *Solyc03g031860.2.1* |
| *Malus domestica* | *Mdo* | *446.0* | *5.5E-154* | *MDP0000151924 (primary)* | *MDP0000151924* | *MDC016098.147:8268..10540 forward* | *Eudicots (rosid)* | *Rosales* | *Solyc03g031860.2.1* |
| *Malus domestica* | *Mdo* | *360.5* | *6,00E-119* | *MDP0000261447 (primary)* | *MDP0000261447* | *MDC019810.234:891..3831 forward* | *Eudicots (rosid)* | *Rosales* | *Solyc03g031860.2.1* |

| Species | Acronymon | Score | Evalue | Transcript Name | CDS Acession Number | Location | Classification group | Order | Query |
|---|---|---|---|---|---|---|---|---|---|
| *Malus domestica* | *Mdo* | *360.5* | *6.5E-119* | *MDP0000321586 (primary)* | *MDP0000321586* | *MDC019810.242:8443..11383 forward* | *Eudicots (rosid)* | *Rosales* | *Solyc03g031860.2.1* |
| *Manihot esculenta* | *Mes* | *634.4* | *0.0* | *Manes.01G124200.1 (primary)* | *Manes.01G124200.1* | *Chromosome01:24153420..24156720 forward* | *Eudicots (rosid)* | *Malpighiales* | *Solyc03g031860.2.1* |
| *Manihot esculenta* | *Mes* | *612.8* | *0.0* | *Manes.02G081700.1 (primary)* | *Manes.02G081700.1* | *Chromosome02:6091016..6093529 forward* | *Eudicots (rosid)* | *Malpighiales* | *Solyc03g031860.2.1* |
| *Manihot esculenta* | *Mes* | *452.6* | *9.8E-157* | *Manes.03G084700.1 (primary)* | *Manes.03G084700.1* | *Chromosome03:13755566..13757622 forward* | *Eudicots (rosid)* | *Malpighiales* | *Solyc03g031860.2.1* |
| *Medicago truncatula* | *Mtr* | *607.1* | *0.0* | *Medtr5g076620.1 (primary)* | *Medtr5g076620.1* | *chr5:32685852..32690189 reverse* | *Eudicots (rosid)* | *Fabales* | *Solyc03g031860.2.1* |
| *Medicago truncatula* | *Mtr* | *546.2* | *0.0* | *Medtr3g450510.1 (primary)* | *Medtr3g450510.1* | *chr3:17379804..17382838 reverse* | *Eudicots (rosid)* | *Fabales* | *Solyc03g031860.2.1* |
| *Medicago truncatula* | *Mtr* | *535.8* | *0.0* | *Medtr5g090780.1 (primary)* | *Medtr5g090780.1* | *chr5:39516910..39526617 reverse* | *Eudicots (rosid)* | *Fabales* | *Solyc03g031860.2.1* |
| *Medicago truncatula* | *Mtr* | *448.7* | *3.6E-155* | *Medtr3g083630.1 (primary)* | *Medtr3g083630.1* | *chr3:37749162..37751789 forward* | *Eudicots (rosid)* | *Fabales* | *Solyc03g031860.2.1* |
| *Arabidopsis halleri* | *Aha* | *602.8* | *0.0* | *Araha.10007s0012.1 (primary)* | *Araha.10007s0012.1* | *Scaffold10007:28956..32226 reverse* | *Eudicots (rosids)* | *Brassicales* | *Solyc03g031860.2.1* |
| *Arabidopsis lyrata* | *Aly* | *599.4* | *0.0* | *AL6G28280.t1 (primary)* | *AL6G28280.t1* | *scaffold_6:7059473..7062737 reverse* | *Eudicots (rosids)* | *Brassicales* | *Solyc03g031860.2.1* |
| *Arabidopsis thaliana* | *Ath* | *604.4* | *0.0* | *AT5G17230.1* | *AT5G17230.1* | *Chr5:5659574..5662865 reverse* | *Eudicots (rosids)* | *Brassicales* | *Solyc03g031860.2.1* |
| *Boechera stricta* | *Bsr* | *606.7* | *0.0* | *Bostr.2618s0058.1 (primary)* | *Bostr.2618s0058.1* | *Scaffold2618:293687..296867 reverse* | *Eudicots (rosids)* | *Brassicales* | *Solyc03g031860.2.1* |
| *Brassica napus* | *Brn* | *630* | *4,00E-179* | *NM_001315554* | *NM_001315554* | *Chr9 NC_027775.2 (44447856..44450852)* | *Eudicots (rosids)* | *Brassicales* | *Solyc03g031860.2.1* |
| *Brassica oleracea* | *Bol* | *589.3* | *0.0* | *Bol019820 (primary)* | *Bol019820* | *C09:32393730..32395844 forward* | *Eudicots (rosids)* | *Brassicales* | *Solyc03g031860.2.1* |
| *Brassica oleracea* | *Bol* | *586.3* | *0.0* | *Bol021326 (primary)* | *Bol021326* | *C02:4117261..4119269 reverse* | *Eudicots (rosids)* | *Brassicales* | *Solyc03g031860.2.1* |
| *Brassica oleracea* | *Bol* | *582.4* | *0.0* | *Bol034439 (primary)* | *Bol034439* | *C03:3362597..3364641 reverse* | *Eudicots (rosids)* | *Brassicales* | *Solyc03g031860.2.1* |
| *Brassica rapa* | *Bra* | *589.0* | *0.0* | *Brara.J01825.1 (primary)* | *Brara.J01825.1* | *A10:14948644..14951545 forward* | *Eudicots (rosids)* | *Brassicales* | *Solyc03g031860.2.1* |
| *Brassica rapa* | *Bra* | *584.7* | *0.0* | *Brara.B00699.1 (primary)* | *Brara.B00699.1* | *A02:3229295..3232410 reverse* | *Eudicots (rosids)* | *Brassicales* | *Solyc03g031860.2.1* |

| Species | Acronymon | Score | Evalue | Transcript Name | CDS Acession Number | Location | Classification group | Order | Query |
|---|---|---|---|---|---|---|---|---|---|
| *Brassica rapa* | *Bra* | *575.5* | *0.0* | *Brara.C00758.1 (primary)* | *Brara.C00758.1* | *A03:3462822..3465336 reverse* | *Eudicots (rosids)* | *Brassicales* | *Solyc03g031860.2.1* |
| *Capsella grandiflora* | *Cgr* | *600.9* | *0.0* | *Cagra.4849s0011.1 (primary)* | *Cagra.4849s0011.1* | *Scaffold4849:47602..50896 forward* | *Eudicots (rosids)* | *Brassicales* | *Solyc03g031860.2.1* |
| *Capsella rubella* | *Cru* | *601.7* | *0.0* | *Carubv10001034m (primary)* | *Carubv10001034m* | *scaffold_6:5698892..5702297 reverse* | *Eudicots (rosids)* | *Brassicales* | *Solyc03g031860.2.1* |
| *Carica papaya* | *Cpa* | *274.2* | *2.9E-90* | *evm.model.supercontig_119.76 (primary)* | *evm.model.supercontig_119.76* | *supercontig_119:492097..492902 forward* | *Eudicots (rosids)* | *Brassicales* | *Solyc03g031860.2.1* |
| *Citrus clementina* | *Ccl* | *620.9* | *0.0* | *Ciclev10011751m* | *Ciclev10011751m* | *scaffold_6:21390477..21396087 reverse* | *Eudicots (rosids)* | *Sapindales* | *Solyc03g031860.2.1* |
| *Citrus clementina* | *Ccl* | *531.9* | *0.0* | *Ciclev10011841m (primary)* | *Ciclev10011841m* | *scaffold_6:21390477..21396087 reverse* | *Eudicots (rosids)* | *Sapindales* | *Solyc03g031860.2.1* |
| *Citrus clementina* | *Ccl* | *443.0* | *4.1E-153* | *Ciclev10018150m (primary)* | *Ciclev10018150m* | *scaffold_2:587065..588778 forward* | *Eudicots (rosids)* | *Sapindales* | *Solyc03g031860.2.1* |
| *Citrus clementina* | *Ccl* | *421.0* | *1.6E-144* | *Ciclev10015582m (primary)* | *Ciclev10015582m* | *scaffold_2:4441285..4443386 forward* | *Eudicots (rosids)* | *Sapindales* | *Solyc03g031860.2.1* |
| *Citrus clementina* | *Ccl* | *313.5* | *5,00E-105* | *Ciclev10018272m (primary)* | *Ciclev10018272m* | *scaffold_2:593780..594691 forward* | *Eudicots (rosids)* | *Sapindales* | *Solyc03g031860.2.1* |
| *Citrus sinensis* | *Csi* | *623.6* | *0.0* | *orange1.1g044623m (primary)* | *orange1.1g044623m* | *scaffold00091:64686..69575 reverse* | *Eudicots (rosids)* | *Sapindales* | *Solyc03g031860.2.1* |
| *Citrus sinensis* | *Csi* | *443.0* | *3.8E-153* | *orange1.1g036368m (primary)* | *orange1.1g036368m* | *scaffold00043:537648..539344 reverse* | *Eudicots (rosids)* | *Sapindales* | *Solyc03g031860.2.1* |
| *Citrus sinensis* | *Csi* | *421.0* | *1.8E-144* | *orange1.1g016696m (primary)* | *orange1.1g016696m* | *scaffold00043:507836..509918 reverse* | *Eudicots (rosids)* | *Sapindales* | *Solyc03g031860.2.1* |
| *Citrus sinensis* | *Csi* | *318.9* | *1.4E-106* | *orange1.1g036976m (primary)* | *orange1.1g036976m* | *scaffold00043:531582..532841 reverse* | *Eudicots (rosids)* | *Sapindales* | *Solyc03g031860.2.1* |
| *Corchorus capsularis* | *Ccp* | *1523* | *0.0* | *OMO59822* | *OMO59822* | *SuperContig contig13869: 10,338-13,426 reverse strand* | *Eudicots (rosids)* | *Malvales* | *Solyc03g031860.2.1* |
| *Corchorus capsularis* | *Ccp* | *1305* | *3,40E-173* | *OMP12065* | *OMP12065* | *SuperContig contig00482: 2,804-5,324 reverse strand* | *Eudicots (rosids)* | *Malvales* | *Solyc03g031860.2.1* |
| *Corchorus capsularis* | *Ccp* | *1134* | *1,80E-149* | *OMO80986* | *OMO80986* | *SuperContig contig10253: 32,456-34,004 forward strand.* | *Eudicots (rosids)* | *Malvales* | *Solyc03g031860.2.1* |
| *Cucumis sativus* | *Csa* | *612.1* | *0.0* | *Cucsa.173570.1 (primary)* | *Cucsa.173570.1* | *scaffold01209:280607..283995 forward* | *Eudicots (rosids)* | *Cucurbitales* | *Solyc03g031860.2.1* |

| Species | Acronymon | Score | Evalue | Transcript Name | CDS Acesion Number | Location | Classification group | Order | Query |
|---|---|---|---|---|---|---|---|---|---|
| *Cucumis sativus* | *Csa* | *538.5* | *0.0* | *Cucsa.179860.1 (primary)* | *Cucsa.179860.1* | *scaffold01233:223487..227034 forward* | *Eudicots (rosids)* | *Cucurbitales* | *Solyc03g031860.2.1* |
| *Cucumis sativus* | *Csa* | *459.5* | *4.8E-161* | *Cucsa.153210.1 (primary)* | *Cucsa.153210.1* | *scaffold01124:412600..414885 forward* | *Eudicots (rosids)* | *Cucurbitales* | *Solyc03g031860.2.1* |
| *Eucalyptus grandis* | *Egr* | *597.4* | *0.0* | *Eucgr.F02913.1 (primary)* | *Eucgr.F02913.1* | *Chr06:41294911..41299376 forward* | *Eudicots (rosids)* | *Myrtales* | *Solyc03g031860.2.1* |
| *Eucalyptus grandis* | *Egr* | *532.3* | *0.0* | *Eucgr.A02030.1 (primary)* | *Eucgr.A02030.1* | *Chr01:35731243..35734193 forward* | *Eudicots (rosids)* | *Myrtales* | *Solyc03g031860.2.1* |
| *Eucalyptus grandis* | *Egr* | *527.3* | *0.0* | *Eucgr.F02914.1 (primary)* | *Eucgr.F02914.1* | *hr06:41301565..41304622 forward* | *Eudicots (rosids)* | *Myrtales* | *Solyc03g031860.2.1* |
| *Eucalyptus grandis* | *Egr* | *455.3* | *1.7E-158* | *Eucgr.B03619.1 (primary)* | *Eucgr.B03619.1* | *Chr02:55924946..55927040 forward* | *Eudicots (rosids)* | *Myrtales* | *Solyc03g031860.2.1* |
| *Eutrema salsugineum* | *Esa* | *593.2* | *0.0* | *Thhalv10013646m* | *Thhalv10013646m* | *scaffold_2:5787369..5790424 reverse* | *Eudicots (rosids)* | *Brassicales* | *Solyc03g031860.2.1* |
| *Eutrema salsugineum* | *Esa* | *585.9* | *0.0* | *Thhalv10013608m (primary)* | *Thhalv10013608m* | *scaffold_2:5787369..5790790 reverse* | *Eudicots (rosids)* | *Brassicales* | *Solyc03g031860.2.1* |
| *Fragaria vesca* | *Fve* | *583.2* | *0.0* | *mrna31674.1-v1.0-hybrid (primary)* | *mrna31674.1-v1.0-hybrid* | *LG1:10143441..10147256 reverse* | *Eudicots (rosids)* | *Rosales* | *Solyc03g031860.2.1* |
| *Fragaria vesca* | *Fve* | *548.9* | *0.0* | *mrna28765.1-v1.0-hybrid (primary)* | *mrna28765.1-v1.0-hybrid* | *LG6:30207289..30209752 forward* | *Eudicots (rosids)* | *Rosales* | *Solyc03g031860.2.1* |
| *Fragaria vesca* | *Fve* | *446.8* | *7.4E-155* | *mrna24795.1-v1.0-hybrid (primary)* | *mrna24795.1-v1.0-hybrid* | *LG3:28382548..28384477 reverse* | *Eudicots (rosids)* | *Rosales* | *Solyc03g031860.2.1* |
| *Glycine max* | *Gma* | *612.5* | *0.0* | *Glyma.02G240200.1 (primary)* | *Glyma.02G240200.1* | *Chr02:42872399..42878147 reverse* | *Eudicots (rosids)* | *Fabales* | *Solyc03g031860.2.1* |
| *Glycine max* | *Gma* | *552.7* | *0.0* | *Glyma.14G209700.1 (primary)* | *Glyma.14G209700.1* | *Chr14:47496019..47500526 reverse* | *Eudicots (rosids)* | *Fabales* | *Solyc03g031860.2.1* |
| *Glycine max* | *Gma* | *548.9* | *0.0* | *Glyma.18G111900.1 (primary)* | *Glyma.18G111900.1* | *Chr18:13308532..13312028 forward* | *Eudicots (rosids)* | *Fabales* | *Solyc03g031860.2.1* |
| *Glycine max* | *Gma* | *547.7* | *0.0* | *Glyma.08G306200.1 (primary)* | *Glyma.08G306200.1* | *Chr08:42422270..42425862 reverse* | *Eudicots (rosids)* | *Fabales* | *Solyc03g031860.2.1* |
| *Glycine max* | *Gma* | *547.4* | *0.0* | *Glyma.14G031200.1 (primary)* | *Glyma.14G031200.1* | *Chr14:2263622..2266628 forward* | *Eudicots (rosids)* | *Fabales* | *Solyc03g031860.2.1* |
| *Glycine max* | *Gma* | *543.5* | *0.0* | *Glyma.02G283400.1 (primary)* | *Glyma.02G283400.1* | *Chr02:46520816..46523824 reverse* | *Eudicots (rosids)* | *Fabales* | *Solyc03g031860.2.1* |

| Species | Acronymon | Score | Evalue | Transcript Name | CDS Acession Number | Location | Classification group | Order | Query |
|---|---|---|---|---|---|---|---|---|---|
| *Glycine max* | *Gma* | *445.3* | *1.2E-153* | *Glyma.18G000600.1 (primary)* | *Glyma.18G000600.1* | *Chr18:99110..101506 forward* | *Eudicots (rosids)* | *Fabales* | *Solyc03g031860.2.1* |
| *Glycine max* | *Gma* | *386.7* | *6.9E-131* | *Glyma.11G256400.1 (primary)* | *Glyma.11G256400.1* | *Chr11:34614957..34616879 reverse* | *Eudicots (rosids)* | *Fabales* | *Solyc03g031860.2.1* |
| *Gossypium raimondii* | *Gra* | *579.3* | *0.0* | *Gorai.001G083700.1 (primary)* | *Gorai.001G083700.1* | *Chr01:8837490..8839782 reverse* | *Eudicots (rosids)* | *Malvales* | *Solyc03g031860.2.1* |
| *Gossypium raimondii* | *Gra* | *547.0* | *0.0* | *Gorai.006G009400.1 (primary)* | *Gorai.006G009400.1* | *Chr06:2095293..2098359 forward* | *Eudicots (rosids)* | *Malvales* | *Solyc03g031860.2.1* |
| *Gossypium raimondii* | *Gra* | *473.0* | *4.7E-165* | *Gorai.012G039100.1 (primary)* | *Gorai.012G039100.1* | *Chr12:4858435..4862016 forward* | *Eudicots (rosids)* | *Malvales* | *Solyc03g031860.2.1* |
| *Gossypium raimondii* | *Gra* | *462.2* | *2.1E-160* | *Gorai.010G126900.1 (primary)* | *Gorai.010G126900.1* | *Chr10:26981829..26983602 forward* | *Eudicots (rosids)* | *Malvales* | *Solyc03g031860.2.1* |
| *Kalanchoe fedtschenko* | *Kfe* | *536.6* | *0.0* | *Kaladp0038s0121.1 (primary)* | *Kaladp0038s0121.1* | *Scaffold_38:972031..974522 reverse* | *Eudicots (rosids)* | *Saxifragales* | *Solyc03g031860.2.1* |
| *Kalanchoe fedtschenko* | *Kfe* | *469.5* | *1.8E-163* | *Kaladp0037s0246.1 (primary)* | *Kaladp0037s0246.1* | *Scaffold_37:1750094..1751770 forward* | *Eudicots (rosids)* | *Saxifragales* | *Solyc03g031860.2.1* |
| *Kalanchoe fedtschenko* | *Kfe* | *330.5* | *9.2E-112* | *Kaladp0673s0002.1 (primary)* | *Kaladp0673s0002.1* | *Scaffold_673:4911..5844 reverse* | *Eudicots (rosids)* | *Saxifragales* | *Solyc03g031860.2.1* |
| *Kalanchoe laxiflora* | *Kla* | *545.4* | *0.0* | *Kalax.0456s0024.1 (primary)* | *Kalax.0456s0024.1* | *scaffold_456:221357..224086 forward* | *Eudicots (rosids)* | *Saxifragales* | *Solyc03g031860.2.1* |
| *Kalanchoe laxiflora* | *Kla* | *542.0* | *0.0* | *Kalax.0207s0024.1 (primary)* | *Kalax.0207s0024.1* | *scaffold_207:338827..341588 reverse* | *Eudicots (rosids)* | *Saxifragales* | *Solyc03g031860.2.1* |
| *Kalanchoe laxiflora* | *Kla* | *538.5* | *0.0* | *Kalax.0551s0002.1 (primary)* | *Kalax.0551s0002.1* | *scaffold_551:16039..18637 reverse* | *Eudicots (rosids)* | *Saxifragales* | *Solyc03g031860.2.1* |
| *Kalanchoe laxiflora* | *Kla* | *533.5* | *0.0* | *Kalax.0033s0007.1 (primary)* | *Kalax.0033s0007.1* | *scaffold_33:58871..61590 reverse* | *Eudicots (rosids)* | *Saxifragales* | *Solyc03g031860.2.1* |
| *Kalanchoe laxiflora* | *Kla* | *468.8* | *6,00E-163* | *Kalax.0026s0034.1 (primary)* | *Kalax.0026s0034.1* | *scaffold_26:332064..333638 forward* | *Eudicots (rosids)* | *Saxifragales* | *Solyc03g031860.2.1* |
| *Kalanchoe laxiflora* | *Kla* | *468.0* | *1.2E-162* | *Kalax.1101s0008.1 (primary)* | *Kalax.1101s0008.1* | *scaffold_1101:71236..72950 forward* | *Eudicots (rosids)* | *Saxifragales* | *Solyc03g031860.2.1* |
| *Phaseolus vulgaris* | *Pvu* | *601.3* | *0.0* | *Phvul.008G241500.1 (primary)* | *Phvul.008G241500.1* | *Chr08:59009458..59014705 forward* | *Eudicots (rosids)* | *Fabales* | *Solyc03g031860.2.1* |
| *Phaseolus vulgaris* | *Pvu* | *553.9* | *0.0* | *Phvul.006G024100.1 (primary)* | *Phvul.006G024100.1* | *Chr06:9727535..9730509 reverse* | *Eudicots (rosids)* | *Fabales* | *Solyc03g031860.2.1* |
| *Phaseolus vulgaris* | *Pvu* | *543.9* | *0.0* | *Phvul.008G195800.1 (primary)* | *Phvul.008G195800.1* | *Chr08:54004623..54007080 forward* | *Eudicots (rosids)* | *Fabales* | *Solyc03g031860.2.1* |

| Species | Acronymon | Score | Evalue | Transcript Name | CDS Acession Number | Location | Classification group | Order | Query |
|---|---|---|---|---|---|---|---|---|---|
| *Phaseolus vulgaris* | *Pvu* | *446.0* | *2.7E-154* | *Phvul.001G268600.1 (primary)* | *Phvul.001G268600.1* | *Chr01:51323835..51325905 reverse* | *Eudicots (rosids)* | *Fabales* | *Solyc03g031860.2.1* |
| *Populus trichocarpa* | *Ptr* | *533.1* | *0.0* | *Potri.005G205800.1 (primary)* | *Potri.005G205800.1* | *Chr05:22042708..22046398 forward* | *Eudicots (rosids)* | *Malpighiales* | *Solyc03g031860.2.1* |
| *Populus trichocarpa* | *Ptr* | *531.2* | *0.0* | *Potri.002G056800.1 (primary)* | *Potri.002G056800.1* | *Chr02:3799091..3802364 reverse* | *Eudicots (rosids)* | *Malpighiales* | *Solyc03g031860.2.1* |
| *Populus trichocarpa* | *Ptr* | *516.5* | *0.0* | *Potri.004G081500.1 (primary)* | *Potri.004G081500.1* | *Chr04:6717614..6720756 forward* | *Eudicots (rosids)* | *Malpighiales* | *Solyc03g031860.2.1* |
| *Populus trichocarpa* | *Ptr* | *498.0* | *9.9E-176* | *Potri.017G138900.1 (primary)* | *Potri.017G138900.1* | *Chr17:14712482..14716973 reverse* | *Eudicots (rosids)* | *Malpighiales* | *Solyc03g031860.2.1* |
| *Populus trichocarpa* | *Ptr* | *452.6* | *1.2E-156* | *Potri.001G007700.1 (primary)* | *Potri.001G007700.1* | *Chr01:518243..520451 forward* | *Eudicots (rosids)* | *Malpighiales* | *Solyc03g031860.2.1* |
| *Populus trichocarpa* | *Ptr* | *424.1* | *1.7E-145* | *Potri.003G218000.1 (primary)* | *Potri.003G218000.1* | *Chr03:21421621..21423998 reverse* | *Eudicots (rosids)* | *Malpighiales* | *Solyc03g031860.2.1* |
| *Prunus avium* | *Pav* | *466* | *1,00E-143* | *Pav_sc0001215.1_g280.1.mk:mrna* | *sc0001215* | *SuperContig Pav_sc0001215.1.: 135,814-138,824 reverse strand* | *Eudicots (rosids)* | *Rosales* | *Solyc03g031860.2.1* |
| *Prunus avium* | *Pav* | *375* | *2,50E-164* | *Pav_sc0001124.1_g480.1.mk:mrna* | *sc0001124* | *SuperContig Pav_sc0001124.1.: 341,607-346,067 reverse strand* | *Eudicots (rosids)* | *Rosales* | *Solyc03g031860.2.1* |
| *Prunus avium* | *Pav* | *335* | *1,90E-99* | *Pav_sc0000909.1_g650.1.mk:mrna* | *sc0000909* | *SuperContig Pav_sc0000909.1.: 406,876-408,851 reverse strand* | *Eudicots (rosids)* | *Rosales* | *Solyc03g031860.2.1* |
| *Prunus persica* | *Ppe* | *631.7* | *0.0* | *Prupe.3G013200.1 (primary)* | *Prupe.3G013200.1* | *Pp03:938428..942844 forward* | *Eudicots (rosids)* | *Rosales* | *Solyc03g031860.2.1* |
| *Prunus persica* | *Ppe* | *553.5* | *0.0* | *Prupe.3G178500.1 (primary)* | *Prupe.3G178500.1* | *Pp03:19498687..19501428 forward* | *Eudicots (rosids)* | *Rosales* | *Solyc03g031860.2.1* |
| *Prunus persica* | *Ppe* | *438.7* | *3,00E-151* | *Prupe.6G006800.1 (primary)* | *Prupe.6G006800.1* | *Pp06:572052..574223 forward* | *Eudicots (rosids)* | *Rosales* | *Solyc03g031860.2.1* |
| *Ricinus communis* | *Rco* | *617.8* | *0.0* | *28611.m000105 (primary)* | *28611.m000105* | *28611:72961..77067 reverse* | *Eudicots (rosids)* | *Malpighiales* | *Solyc03g031860.2.1* |
| *Ricinus communis* | *Rco* | *451.4* | *1.4E-156* | *29835.m000639 (primary)* | *29835.m000639* | *29835:165737..167782 forward* | *Eudicots (rosids)* | *Malpighiales* | *Solyc03g031860.2.1* |
| *Ricinus communis* | *Rco* | *398.7* | *3.9E-136* | *29835.m000640 (primary)* | *29835.m000640* | *29835:168336..171416 reverse* | *Eudicots (rosids)* | *Malpighiales* | *Solyc03g031860.2.1* |
| *Salix purpurea* | *Spu* | *618.6* | *0.0* | *SapurV1A.0549s0100.1 (primary)* | *SapurV1A.0549s0100.1* | *chr17:16798169..16802345 reverse* | *Eudicots (rosids)* | *Malpighiales* | *Solyc03g031860.2.1* |

| Species | Acronymon | Score | Evalue | Transcript Name | CDS Acession Number | Location | Classification group | Order | Query |
|---|---|---|---|---|---|---|---|---|---|
| *Salix purpurea* | *Spu* | *538.5* | *0.0* | *SapurV1A.1979s0010.1 (primary)* | *apurV1A.1979s0010.1* | *chr05:17477652..17480791 forward* | *Eudicots (rosids)* | *Malpighiales* | *Solyc03g031860.2.1* |
| *Salix purpurea* | *Spu* | *537.0* | *0.0* | *SapurV1A.0076s0100.1 (primary)* | *SapurV1A.0076s0100.1* | *chr05:17594349..17597440 forward* | *Eudicots (rosids)* | *Malpighiales* | *Solyc03g031860.2.1* |
| *Salix purpurea* | *Spu* | *535.0* | *0.0* | *SapurV1A.4120s0010.1 (primary)* | *SapurV1A.4120s0010.1* | *Scaffold4120:6004..8854 forward* | *Eudicots (rosids)* | *Malpighiales* | *Solyc03g031860.2.1* |
| *Salix purpurea* | *Spu* | *529.6* | *0.0* | *SapurV1A.0354s0270.1 (primary)* | *SapurV1A.0354s0270.1* | *chr15:13280059..13282909 reverse* | *Eudicots (rosids)* | *Malpighiales* | *Solyc03g031860.2.1* |
| *Salix purpurea* | *Spu* | *405.2* | *4.4E-138* | *SapurV1A.0081s0270.1 (primary)* | *SapurV1A.0081s0270.1* | *chr03:16337996..16340689 reverse* | *Eudicots (rosids)* | *Malpighiales* | *Solyc03g031860.2.1* |
| *Theobroma cacao* | *Tca* | *633.3* | *0.0* | *Thecc1EG017615t1 (primary)* | *Thecc1EG017615t1* | *scaffold_4:5316486..5321472 forward* | *Eudicots (rosids)* | *Malvales* | *Solyc03g031860.2.1* |
| *Theobroma cacao* | *Tca* | *552.7* | *0.0* | *Thecc1EG001945t1 (primary)* | *Thecc1EG001945t1* | *scaffold_1:10439470..10442560 reverse* | *Eudicots (rosids)* | *Malvales* | *Solyc03g031860.2.1* |
| *Theobroma cacao* | *Tca* | *459.5* | *1.1E-159* | *Thecc1EG040196t1 (primary)* | *Thecc1EG040196t1* | *scaffold_9:27850549..27853174 forward* | *Eudicots (rosids)* | *Malvales* | *Solyc03g031860.2.1* |
| *Vigna radiata* | *Vra* | *476* | *9,80E-136* | *Vradi01g13040.1* | *Vradi01g13040.1* | *Chromosome 1: 29,983,650-29,986,312 forward strand* | *Eudicots (rosids)* | *Fabales* | *Solyc03g031860.2.1* |
| *Vigna radiata* | *Vra* | *459* | *2,10E-129* | *Vradi06g02420.1* | *Vradi06g02420.1* | *Chromosome 6: 2,396,136-2,398,470 forward strand.* | *Eudicots (rosids)* | *Fabales* | *Solyc03g031860.2.1* |
| *Vigna radiata* | *Vra* | *379* | *1,30E-146* | *Vradi06g05990.1* | *Vradi06g05990.1* | *Chromosome 6: 7,578,171-7,583,722 forward strand.* | *Eudicots (rosids)* | *Fabales* | *Solyc03g031860.2.1* |
| *Vigna radiata* | *Vra* | *334* | *4,30E-102* | *Vradi0246s00050.1* | *Vradi0246s00050.1* | *SuperContig scaffold_246: 120,684-123,023 forward strand* | *Eudicots (rosids)* | *Fabales* | *Solyc03g031860.2.1* |
| *Vitis vinifera* | *Vvi* | *664.8* | *0.0* | *GSVIVT01035255001* | *GSVIVT01035255001* | *chr4:11494435..11499618 forward* | *Eudicots (rosids)* | *Vitales* | *Solyc03g031860.2.1* |
| *Vitis vinifera* | *Vvi* | *510.4* | *0.0* | *GSVIVT01020828001* | *GSVIVT01020828001* | *chr12:1467463..1470527 forward* | *Eudicots (rosids)* | *Vitales* | *Solyc03g031860.2.1* |
| *Vitis vinifera* | *Vvi* | *451.8* | *3,00E-157* | *GSVIVT01025421001* | *GSVIVT01025421001* | *chr6:935380..940685 forward* | *Eudicots (rosids)* | *Vitales* | *Solyc03g031860.2.1* |
| *Beta vulgaris* | *Bvu* | *392* | *7,00E-143* | *KMT13325* | *KMT13325* | *Chromosome 4: 20,504,431-20,508,295 reverse strand* | *Eudicots (superasterids)* | *Caryophyllales* | *Solyc03g031860.2.1* |
| *Trifolium pratense* | *Tpr* | *602.8* | *0.0* | *Tp57577_TGAC_v2_mRNA5500 (primary)* | *Tp57577_TGAC_v2_mRNA5500* | *Tp57577_TGAC_v2_LG2:23973554..23978020 forward* | *Eudicots(rosids)* | *Fabales* | *Solyc03g031860.2.1* |

| Species | Acronymon | Score | Evalue | Transcript Name | CDS Acession Number | Location | Classification group | Order | Query |
|---|---|---|---|---|---|---|---|---|---|
| *Trifolium pratense* | *Tpr* | *537.7* | *0.0* | *Tp57577_TGAC_v2_mRNA26125 (primary)* | *Tp57577_TGAC_v2_mRNA26125* | *Tp57577_TGAC_v2_LG5:14102402..14106056 forward* | *Eudicots(rosids)* | *Fabales* | *Solyc03g031860.2.1* |
| *Trifolium pratense* | *Tpr* | *460.3* | *5.8E-160* | *Tp57577_TGAC_v2_mRNA19990 (primary)* | *Tp57577_TGAC_v2_mRNA19990* | *Tp57577_TGAC_v2_scaf_333:243896..246365 forward* | *Eudicots(rosids)* | *Fabales* | *Solyc03g031860.2.1* |
| *Trifolium pratense* | *Tpr* | *443,7* | *1.6E-152* | *Tp57577_TGAC_v2_mRNA9064 (primary)* | *Tp57577_TGAC_v2_mRNA9064* | *Tp57577_TGAC_v2_scaf_512:4326..10060 forward* | *Eudicots(rosids)* | *Fabales* | *Solyc03g031860.2.1* |
