# Supplementary material for: Molecular evolution and diversification of phytoene synthase (PSY) gene family
Source: Genet Mol Biol. 2022 Dec 19;45(4):e20210411. doi: 10.1590/1678-4685-GMB-2021-0411 (PMC9764326; doi:10.1590/1678-4685-GMB-2021-0411)
Supplement: Figure S7 - [file 1415-4757-GMB-45-4-e20210411-s8.pdf]

Genetics and Molecular Biology

https://doi.org/10.1590/1678-4685-GMB-2021-0411

Supplementary material to "Molecular evolution and diversification of phytoene synthase (PSY) gene family"

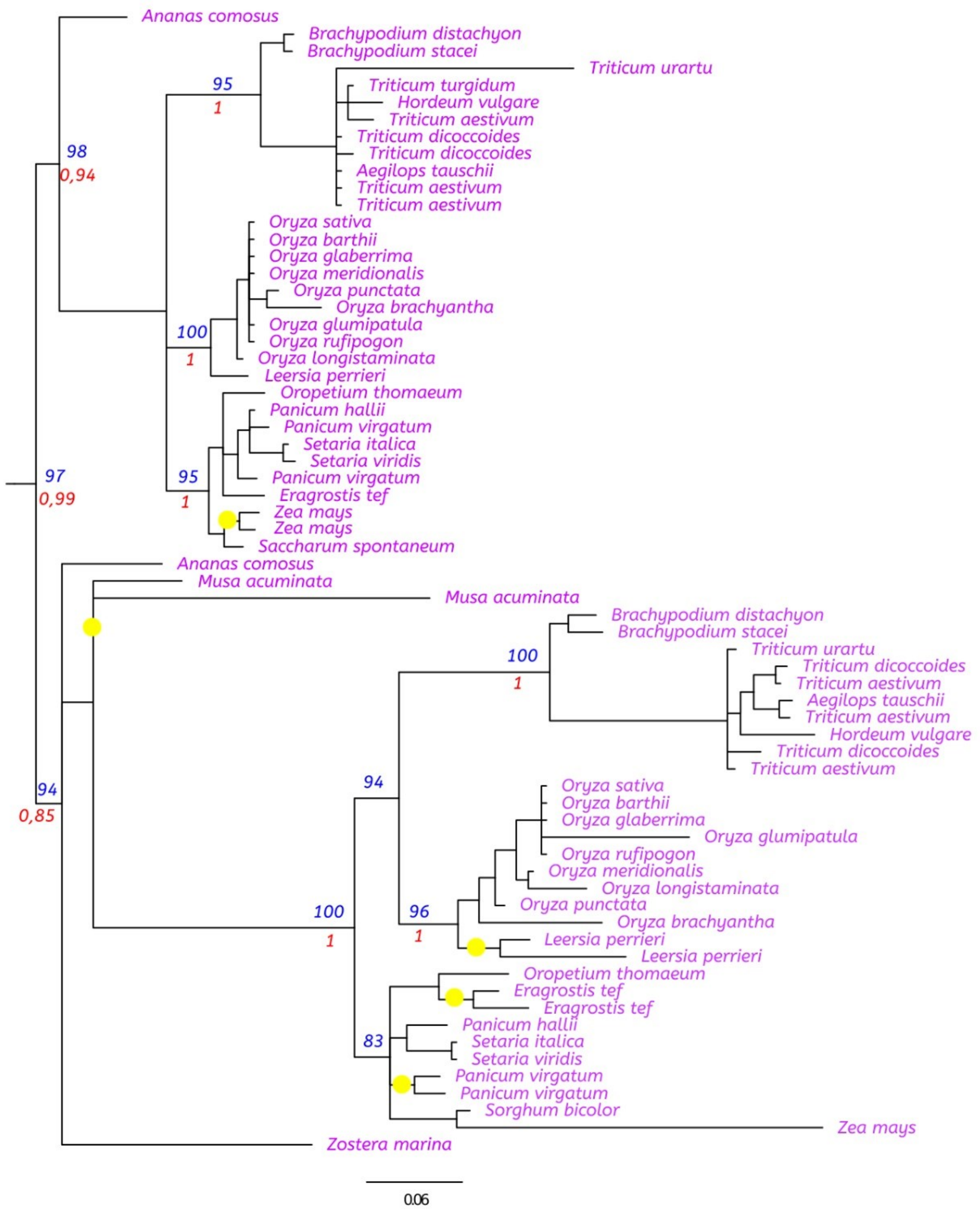

Figure S7 - Phylogenetic relationships among PSY genes belonging to Subgroup M2 from Figure 1 presents both posterior probability (numbers in red) with a cut-off point of 0,8 and bootstrap values (numbers in blue) with a cut-off point of 70. Yellow circle represents the duplication events. The complete list of species is presented in Table S1.
