# Supplementary material for: Molecular evolution and diversification of phytoene synthase (PSY) gene family
Source: Genet Mol Biol. 2022 Dec 19;45(4):e20210411. doi: 10.1590/1678-4685-GMB-2021-0411 (PMC9764326; doi:10.1590/1678-4685-GMB-2021-0411)
Supplement: Figure S9 - [file 1415-4757-GMB-45-4-e20210411-s10.pdf]

Genetics and Molecular Biology

https://doi.org/10.1590/1678-4685-GMB-2021-0411

Supplementary material to "Molecular evolution and diversification of phytoene synthase (PSY) gene family"

0% 100%

Percent of Expression Potential

Arabidopsis thaliana (127)

	PSY1	samples	avg. expr.
callus		31	11.49
▼ cell culture / primary cell		714	11.53
sperm cell		3	13.49
▼ protoplast		287	10.83
▼ root protoplast		236	10.51
▼ root cap protoplast		21	10.47
columella protoplast		18	10.34
lateral root cap protoplast		3	11.22
root epidermis and lateral root cap protoplast		43	10.33
root cortex protoplast		38	11.23
root endodermis and quiescent center protoplast		37	10.50
▼ root stele protoplast		72	10.43
▼ root phloem protoplast		7	10.46
root protophloem and metaphloem protoplast		2	9.73
root phloem companion cell protoplast		3	10.90
root xylem protoplast		6	9.18
▼ root pericycle protoplast		11	11.12
root phloem pole pericycle protoplast		2	12.43
root xylem pole pericycle protoplast		9	10.83
root cortex, endodermis and quiescent center protoplast		2	10.56
▼ root epidermis protoplast		4	10.07
root epidermal atrichoblast protoplast		2	10.53
root hair cell protoplast		2	9.61
lateral root primordium protoplast		2	12.42
▼ leaf protoplast		39	11.95
guard cell protoplast		27	11.88
mesophyll cell protoplast		4	13.30
▼ root cell		48	10.58
root cortex cell		8	11.05
root epid. atrichoblast and phloem companion cell		5	10.39
root endodermis and quiescent center cell		4	10.35
▼ root stele cell		14	10.58
▼ root vascular tissue cell		10	10.49
▼ root phloem cell		6	10.37
root phloem companion cell		6	10.37
root culture		18	11.81
seedling culture		65	13.53
giant cell		9	10.22
▼ shoot cell		68	12.66
▼ shoot epidermis cell		12	13.37
cotyledon and leaf pavement cell		4	12.71
trichome and leaf petiole epidermis cell		4	13.31
cotyledon and leaf guard cell		4	14.09
▼ shoot stele cell		4	13.77
▼ shoot vascular tissue cell		4	13.77
▼ shoot phloem cell		4	13.77
shoot phloem companion cell		4	13.77
shoot vascular tissue and bundle sheath cell		2	13.94
rosette cell		6	14.20
▼ inflorescence stem cell		18	10.73
▼ internode cell		18	10.73
▼ cortex cell		7	11.19
starch sheath (endodermis) cell		7	11.19
interfascicular cambium cell		3	10.25

leaf cell	18	12.67
leaf epidermis cell	18	12.67
guard cell	18	12.67
seedling	2331	13.59
cotyledon	28	14.52
shoot apex	25	13.35
shoot apical meristem	17	13.95
hypocotyl	27	12.57
radicle	23	11.00
inflorescence	801	12.36
raceme	327	12.82
flower	324	12.81
stamen	118	11.52
anther	52	9.84
pollen	52	9.84
abscission zone	15	13.15
pistil	40	13.07
carpel	11	13.13
stigma	3	14.30
ovary	6	12.76
ovule	2	11.74
petal	6	14.41
sepal	6	14.24
pedicel	3	14.77
silique	372	11.73
seed	323	11.47
embryo	40	11.63
suspensor	2	9.83
endosperm	41	12.14
micropylar endosperm	8	11.32
nonmicropylar endosperm	22	12.48
peripheral endosperm	10	12.38
chalazal endosperm	12	12.56
testa	25	10.61
general seed coat	8	10.62
chalazal seed coat	13	10.76
pericarp	7	13.16
replum	7	13.16
shoot	4606	14.06
inflorescence stem	91	13.57
node	3	14.02
internode	3	10.20
stele	3	10.20
conducting tissue	3	10.20
phloem	3	10.20
developing meristemoid zone	3	12.90
rosette	3904	14.13
stem	3	13.80
leaf	2845	14.08
petiole	24	13.68
juvenile leaf	190	14.31
adult leaf	1006	13.94
senescent leaf	3	13.69
axillary bud	2	14.04
cauline leaf	3	14.91
shoot apex	255	13.44
shoot apical meristem	71	13.95
leaf primordia	11	14.87
axillary shoot	2	13.49
hypocotyl	42	12.19
stele	9	12.63
conducting tissue	9	12.63
xylem	9	12.63
cork	9	11.56
roots	1081	10.55
primary root	235	10.38
root tip	102	10.26
root apical meristem	17	9.78
elongation zone	66	10.12
maturation zone	33	10.68
stele	5	12.12
pericycle	5	12.12
lateral root	43	11.22

Figure S9 - Gene expression analysis across anatomical parts of A. thaliana performed using GENEVESTIGATOR.
