# Supplementary material for: Molecular evolution and diversification of phytoene synthase (PSY) gene family
Source: Genet Mol Biol. 2022 Dec 19;45(4):e20210411. doi: 10.1590/1678-4685-GMB-2021-0411 (PMC9764326; doi:10.1590/1678-4685-GMB-2021-0411)
Supplement: Figure S10 - [file 1415-4757-GMB-45-4-e20210411-s11.pdf]

Genetics and Molecular Biology

https://doi.org/10.1590/1678-4685-GMB-2021-0411

Supplementary material to "Molecular evolution and diversification of phytoene synthase (PSY) gene family"

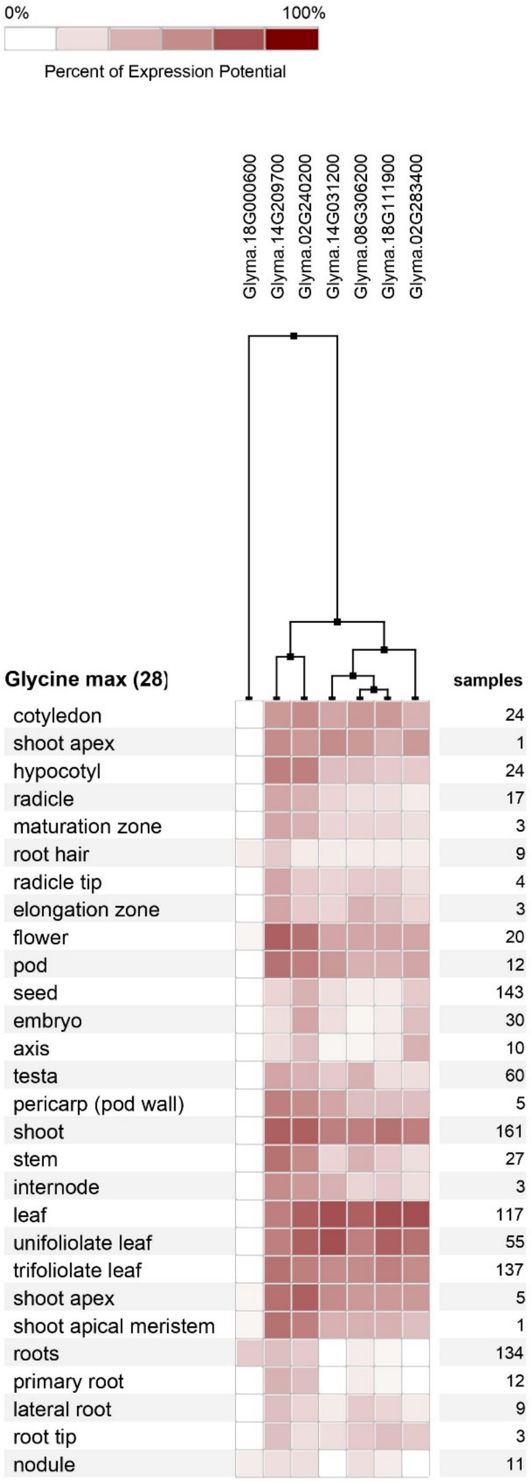

Figure S10 - Gene expression analysis across anatomical parts of G. max performed using GENEVESTIGATOR.
